# Dynamic Public Health Surveillance to Track and Mitigate the US COVID-19 Epidemic: Longitudinal Trend Analysis Study

**DOI:** 10.2196/24286

**Published:** 2020-12-03

**Authors:** Lori Ann Post, Tariq Ziad Issa, Michael J Boctor, Charles B Moss, Robert L Murphy, Michael G Ison, Chad J Achenbach, Danielle Resnick, Lauren Nadya Singh, Janine White, Joshua Marco Mitchell Faber, Kasen Culler, Cynthia A Brandt, James Francis Oehmke

**Affiliations:** 1 Buehler Center for Health Policy & Economics and Departments of Emergency Medicine Feinberg School of Medicine Northwestern University Chicago, IL United States; 2 Feinberg School of Medicine Northwestern University Chicago, IL United States; 3 Institute of Food and Agricultural Sciences University of Florida Gainesville, FL United States; 4 Center for Global Communicable Diseases Institute for Global Health Northwestern University Chicago, IL United States; 5 Divsion of Infectious Disease Feinberg School of Medicine Northwestern University Chicago, IL United States; 6 International Food Policy Research Institute Washington, DC United States; 7 Yale Center for Medical Informatics Yale School of Medicine Yale University New Haven, CT United States

**Keywords:** global COVID-19 surveillance, United States public health surveillance, US COVID-19, surveillance metrics, dynamic panel data, generalized method of the moments, United States econometrics, US SARS-CoV-2, US COVID-19 surveillance system, US COVID-19 transmission speed, COVID-19 transmission acceleration, COVID-19 speed, COVID-19 acceleration, COVID-19 jerk, COVID-19 persistence, Arellano-Bond estimator, COVID-19

## Abstract

**Background:**

The emergence of SARS-CoV-2, the virus that causes COVID-19, has led to a global pandemic. The United States has been severely affected, accounting for the most COVID-19 cases and deaths worldwide. Without a coordinated national public health plan informed by surveillance with actionable metrics, the United States has been ineffective at preventing and mitigating the escalating COVID-19 pandemic. Existing surveillance has incomplete ascertainment and is limited by the use of standard surveillance metrics. Although many COVID-19 data sources track infection rates, informing prevention requires capturing the relevant dynamics of the pandemic.

**Objective:**

The aim of this study is to develop dynamic metrics for public health surveillance that can inform worldwide COVID-19 prevention efforts. Advanced surveillance techniques are essential to inform public health decision making and to identify where and when corrective action is required to prevent outbreaks.

**Methods:**

Using a longitudinal trend analysis study design, we extracted COVID-19 data from global public health registries. We used an empirical difference equation to measure daily case numbers for our use case in 50 US states and the District of Colombia as a function of the prior number of cases, the level of testing, and weekly shift variables based on a dynamic panel model that was estimated using the generalized method of moments approach by implementing the Arellano-Bond estimator in R.

**Results:**

Examination of the United States and state data demonstrated that most US states are experiencing outbreaks as measured by these new metrics of speed, acceleration, jerk, and persistence. Larger US states have high COVID-19 caseloads as a function of population size, density, and deficits in adherence to public health guidelines early in the epidemic, and other states have alarming rates of speed, acceleration, jerk, and 7-day persistence in novel infections. North and South Dakota have had the highest rates of COVID-19 transmission combined with positive acceleration, jerk, and 7-day persistence. Wisconsin and Illinois also have alarming indicators and already lead the nation in daily new COVID-19 infections. As the United States enters its third wave of COVID-19, all 50 states and the District of Colombia have positive rates of speed between 7.58 (Hawaii) and 175.01 (North Dakota), and persistence, ranging from 4.44 (Vermont) to 195.35 (North Dakota) new infections per 100,000 people.

**Conclusions:**

Standard surveillance techniques such as daily and cumulative infections and deaths are helpful but only provide a static view of what has already occurred in the pandemic and are less helpful in prevention. Public health policy that is informed by dynamic surveillance can shift the country from reacting to COVID-19 transmissions to being proactive and taking corrective action when indicators of speed, acceleration, jerk, and persistence remain positive week over week. Implicit within our dynamic surveillance is an early warning system that indicates when there is problematic growth in COVID-19 transmissions as well as signals when growth will become explosive without action. A public health approach that focuses on prevention can prevent major outbreaks in addition to endorsing effective public health policies. Moreover, subnational analyses on the dynamics of the pandemic allow us to zero in on where transmissions are increasing, meaning corrective action can be applied with precision in problematic areas. Dynamic public health surveillance can inform specific geographies where quarantines are necessary while preserving the economy in other US areas.

## Introduction

### Background

**Figure 1:**
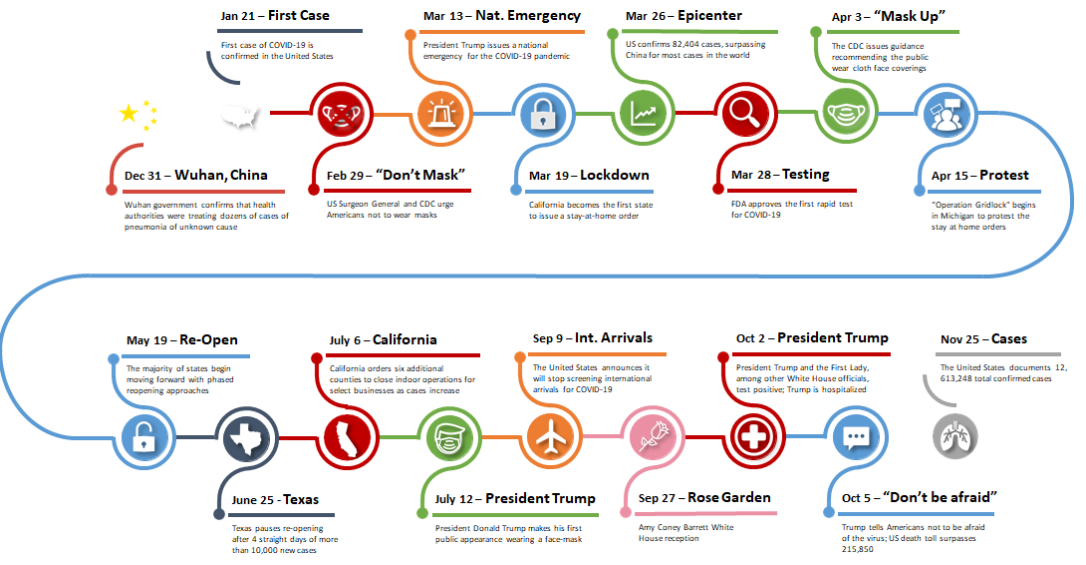
Timeline of COVID-19 in the United States. CDC: Centers for Disease Control and Prevention; FDA: Food and Drug Administration.

The emergence of SARS-CoV-2 in 2019 led to its associated disease, COVID-19, becoming one of the most severe and widespread disease outbreaks in modern history [1]. On January 21, 2020, the first confirmed case of COVID-19 was recorded in the United States (see Figure 1) [2]. Over the next 2 months, cases spread worldwide at an alarming rate, leading the World Health Organization on March 11, 2020, to officially recognize COVID-19 as a pandemic [3]. Around the world, countries quickly implemented public health policies to mitigate the public health and economic impacts caused by the virus. Nations have had varying levels of success at controlling the transmission of COVID-19 due to numerous political, cultural, economic, and structural factors [4]. By November 13, 2020, the daily number of new COVID-19 cases in the United States reached 177,224, the highest number for any single day recorded since the pandemic began, contributing to the 11,485,176 cumulative cases of people infected by COVID-19 and the 250,029 people who have died from it [5].

It is logical that the United States experienced technical difficulties at the beginning of the pandemic because we were dealing with a new virus, a pandemic unprecedented in modern times, and uncharted territory [6], which explains why the United States became the third global epicenter of the pandemic due to a delayed response [7]. It is illogical that the United States continues to underperform every country worldwide in controlling COVID-19 because of our wealth of resources and that we now know the epidemiological risk factors, cultural norms, and politicized public health policies that result in case clustering in the United States [4]. Unfortunately, the current US response to COVID-19 parallels President Wilson’s response to the 1918 Influenza Pandemic that killed 675 million Americans because of no national response to control the outbreak, misinformation, unclear communication with the public, and widespread mistrust and panic [8-13].

### Public Health Policies and Governance

Without a national plan to control COVID-19, some mayors and governors implemented policies such as public masking mandates, prohibitions on gatherings, school closings, restrictions on commercial activities, and broad social distancing measures in an attempt to “flatten the curve” [1,14,15]. Empirical evidence supports social distancing, quarantines, and wearing masks as a means to stop the transmission of COVID-19 [14,16-18]. Leaders who minimize the pandemic, such as White House Press Secretary Kayleigh McEnany, who stated that rising cases are nothing more than “embers that need to be put out” [19], have fostered a false optimism bias that undermines public health guidelines [20]. Framing adherence to those guidelines as a civil liberties issue discourages safe practices, such as advisories by Trump coronavirus advisor Scott Atlas, who urged Michigan to *rise up* against new COVID-19 policies put in place by Governor Whitmer [21]. Conflicting messaging and politicization of COVID-19 have fueled the pandemic. Although the United States only comprises 4% of the global population, it exceeds 20% of the global COVID-19 caseload [22]. Presently, the world is implementing policies to control the second wave of COVID-19, while the United States is responding to its third wave [23-25].

Research has explained why the Unites States consistently leads the world in COVID-19 cases and how we lost control of the outbreak; however, what has not been widely recognized is the need for ongoing systematic public health surveillance to gain control of the pandemic [26-28]. Surveillance informs prevention efforts and signals whether policies put in place to combat COVID-19 are effective or where outbreaks are occurring unabated [26,27]. There are several excellent sources of surveillance data that enumerate the daily and cumulative number of new infections and deaths, the rates of infections and deaths, as well as moving 7-day averages of infections and deaths [29]. Although these indicators are useful, they also have incomplete case ascertainment [26-28]. Without universal testing, surveillance systems are limited to only those cases that are reported from those who are tested, hence erring toward individuals with more severe symptoms [26-28]. Moreover, traditional surveillance metrics are static and provide a proxy of the pandemic for what already has occurred while omitting the pandemics’ dynamics that will inform future effective public health guidelines at the epicenter of outbreaks [30]. Dynamic surveillance allows public health leaders to be proactive rather than reactive, which can prevent novel infections. Without universal testing and a national plan to control the pandemic, public health surveillance is even more essential. Moreover, those leaders concerned with choosing between protecting the public from COVID-19 versus protecting the economy require dynamic public health surveillance to understand which parts of each state can remain open and which must go into quarantine [31-34].

### Objective

The objective of this paper is to use static and dynamic surveillance metrics to measure the caseload and dynamics of the COVID-19 pandemic at the national and state level [30,35-38]. The basic question we are trying to inform is: how are we doing this week relative to previous weeks? From a public health perspective, we need to understand if there are more cases per day this week than last week, the number of new cases is increasing from day to day, and the number of day-to-day increases in the number of cases is bigger this week than last week. Additionally, we would like some indicative information about significant shifts in how the pandemic is progressing—positive shifts could be the first indicators of the emergence of a new or recurrent hot spot, and negative shifts could be the first indicators of a successful public health policy.

## Methods

This study relies on a longitudinal trend analysis study design to investigate the transmission of COVID-19 at the national and the state level over time. The COVID Tracking Project [39] compiles data found on the web [40] from public health departments, universities, and the media to track COVID-19 [3,29,41-43]. Data for the past several months were accessed from the GitHub repository [44]. This resulted in a balanced panel of all 50 states and the District of Columbia (51 x 46 days = 2346). There were in total 3927 observations used to estimate 46 days of data for US states in this analysis. An empirical difference equation was specified in which the number of positive cases in each state at each day is a function of the prior number of cases, the level of testing, and weekly shift variables that measure whether the contagion was growing faster, the same, or slower than the previous weeks. This resulted in a dynamic panel model that was estimated using the generalized method of moments approach by implementing the Arellano-Bond estimator in R (The R Foundation for Statistical Computing) [45,46].

Arellano-Bond estimation of difference equations has several statistical advantages: it allows for statistical examination of the model’s predictive ability and the validity of the model specification, it corrects for autocorrelation and heteroscedasticity, it has good properties for data with a small number of time periods and large number of states, and it corrects for omitted variables issues and provides a statistical test of correction validity. With these advantages, the method is applicable to ascertaining and statistically validating changes in the evolution of the pandemic within a period of a week or less, such as changes in the reproduction rate. Oehmke et al [28,30] provide a detailed discussion of the methods. Finally, we calculated these indicators to inform public health leaders where to take corrective action at a local level.

To ascertain whether pandemic growth was explosive, we examined whether the acceleration and jerk were positive and if the persistence effect was positive as well as if it was larger than the speed. We examined these indicators week over week for 7 weeks along with the daily caseload. The persistence effect is an indicator for mathematically explosive growth (ie, it indicates if the difference equation has a solution that lies outside the unit circle), but large positive acceleration and jerk as well as persistence are indicative of explosive growth in a practical sense (ie, the COVID-19 caseload is expected to increase much more rapidly than in the current or recent week). However, this is a forward-looking statement and must be interpreted with due circumspection. To discuss potential explosive growth, we looked at five data points including speed, acceleration, jerk, persistence, and daily caseload each week for 7 weeks.

## Results

### State Regression Results

We present regression results for the panel comprising all 50 US states and the District of Columbia in Table 1. The subsequent weekly surveillance products were based on these regressions.

**Table 1:** Arellano-Bond dynamic panel data model of COVID-19 dynamics at the state level.

Variable	Values	*P* value
7-day lag, coefficient	1.09	<.001
Cumulative tests, coefficient	0.01	.48
7-day lag shift, coefficient	0.108	<.03
Wald statistic for regression, chi-square (*df*)	13,504 (8)	<.001
Sargan statistic for validity, chi-square (*df*)	48.3 (367)	>.99

The regression Wald statistic was statistically significant (χ^2^_8_=13,504, *P*<.001). The Sargan test was insignificant, failing to reject the validity of overidentifying restrictions (χ^2^_367_=48.3, *P*>.99).

The coefficients on the 7-day lag were both positive and statistically significant (*P*<.001), demonstrating that the number of infections 1 week prior to data collection had a significant effect on number of infections at the point of data collection. Cumulative tests also had a slightly positive but insignificant effect (coefficient 0.01, *P*=.48). The shift parameter 14 days ago was positive and significant (coefficient 0.108, *P*<.03), suggesting that exogenous shift events had a positive effect on cases.

### Interpretation: Regression Results

The lagging indicators and shift parameters suggested recent change in disease transmission in the United States between September 28 and November 15, 2020. Specifically, the most recent 7-day lag this week was 10% faster than last week. The shift in the most recent 14 days, or 2 weeks, was positive and significant (*P*<.03).

### Surveillance Results

Table 2 demonstrates the dynamics of the pandemic for the leading six states in speed, acceleration, jerk, 7-day persistence, and 7-day moving average over the course of the 7-week study beginning on September 28 and ending November 15, 2020. Table 2 is truncated and only lists the leading six states for our metrics. For a complete view of each state, Multimedia Appendix 1 contains Tables S5-S18 that list the traditional surveillance metrics as well as the dynamic surveillance metrics of speed, acceleration, and 7-day persistence rate.

**Table 2:** State pandemic dynamics

Date, State		Speed	State	Acceleration	State	Jerk	State	7-day persistence effect	State	7-day moving average
**Oct 1, 2020**										
	ND	51.9	SD	4.6	SD	5.4	ND	55.7	TX	4083.1
	SD	49.1	AK	2.6	AK	1.6	SD	42.4	CA	3292.4
	WI	42.7	ID	1.7	MO	1.5	WI	37.7	WI	2489.0
	UT	30.9	MT	1.3	ID	1.2	UT	31.4	FL	2250.7
	MT	30.2	WI	1.3	TN	1.0	OK	31.2	NC	2102.9
	AR	27.3	MO	1.0	NV	0.8	AR	30.1	IL	2051.4
**Oct 8, 2020**										
	ND	59.4	ND	2.9	VA	2.7	ND	56.5	TX	4184.7
	SD	52.9	MT	2.5	ND	2.0	SD	53.4	CA	3016.1
	WI	42.6	VA	2.3	IA	1.8	WI	46.6	WI	2477.7
	MT	42.5	UT	2.2	UT	1.7	UT	33.6	FL	2363.7
	UT	35.2	SC	1.9	RI	1.6	MT	32.9	IL	2215.7
	ID	29.6	TN	1.5	NJ	1.4	AR	29.8	NC	1784.0
**Oct 15, 2020**										
	ND	80.0	SD	4.7	SD	7.2	ND	64.6	TX	4002.0
	SD	74.3	MS	3.6	MS	6.7	SD	57.6	CA	3371.6
	MT	56.9	ND	3.4	LA	4.6	WI	46.3	WI	3093.6
	WI	53.1	NE	2.1	KY	4.4	MT	46.2	IL	3031.3
	UT	37.9	WI	1.9	AK	3.1	UT	38.3	FL	2648.1
	NE	37.4	AL	1.8	MT	3.0	ID	32.3	NC	1934.6
**Oct 22, 2020**										
	ND	101.2	AL	3.9	SD	7.5	ND	87.1	TX	5041.7
	SD	80.8	ND	3.4	MO	7.4	SD	80.9	IL	4155.4
	MT	62.9	SD	2.8	ND	7.2	MT	61.9	WI	3528.7
	WI	60.6	MT	2.7	AL	6.2	WI	57.9	FL	3231.6
	ID	46.0	ID	2.7	MT	2.5	UT	41.3	CA	3189.0
	NE	43.1	RI	2.7	FL	1.9	NE	40.8	TN	2154.3
**Oct 29, 2020**										
	ND	113.9	ND	6.3	WI	4.3	ND	110.2	TX	5960.0
	SD	112.8	WI	3.7	CT	3.0	SD	88.0	IL	5203.6
	WI	75.8	MN	3.3	WY	1.8	MT	68.5	CA	4597.1
	MT	69.7	CT	3.3	NV	1.3	WI	66.0	WI	4411.7
	WY	58.9	MO	2.8	MO	1.2	ID	50.1	FL	3717.9
	AK	51.7	MI	2.7	MN	1.2	NE	47.0	MI	2852.4
**Nov 5, 2020**										
	ND	163.2	IA	9.3	SD	11.2	ND	134.6	IL	7654.1
	SD	131.6	ND	5.9	IA	7.0	SD	132.9	TX	6882.0
	WI	90.5	SD	5.8	MS	4.2	WI	89.5	WI	5266.6
	MT	81.7	NE	4.9	RI	2.4	MT	82.1	FL	4577.9
	WY	71.6	UT	4.3	IL	2.4	WY	69.4	CA	4524.6
	IA	71.3	IL	4.0	KY	2.2	AK	61.2	MI	4031.4
**Nov 12, 2020**										
	ND	175.0	WY	18.3	WY	25.5	ND	195.3	IL	11,827.4
	SD	154.5	SD	10.7	LA	12.8	SD	157.5	TX	8406.7
	WY	125.1	LA	9.7	ND	6.5	WI	108.3	CA	6719.0
	WI	112.9	MN	8.3	MN	5.6	MT	97.8	WI	6571.6
	IA	112.6	UT	5.0	UT	4.0	WY	85.7	MI	5845.7
	NE	103.8	ND	4.9	SD	3.8	IA	85.3	OH	5612.4

Although Texas and California ranked in the top six states for daily average of new infections for 7 consecutive weeks, they did not rank high for rates of speed, acceleration, jerk, or 7-day persistence. Florida ranked in the top for daily average of new infections for 6 of the 7 weeks, and in the middle of the study during the week of October 22, 2020, Florida had a positive jerk of 1.9 per 100,000 people, indicating that the pandemic was not just accelerating but accelerating at an increasing rate. North Carolina ranked in the top six states for novel infections for 3 of the 6 weeks but did not rank high for rates of speed, acceleration, jerk, or persistence. Illinois was ranked in the top six states for novel infections all 7 weeks; during the week of November 5, Illinois ranked high in acceleration at 4.0 additional new daily infections per 100,000 people per day, or 28 per week, and had a positive jerk of 2.4 infections per 100,000 people during the same week, indicating that acceleration increased from 1.6 in the prior week to 4.0 the week of November 5.

In Table 2, we examine speed, acceleration, jerk, persistence, and caseload each week for 7 weeks for the top six states for each metric. Dynamic metrics for North Dakota during the 7-week study were alarming with 22 positive data points that indicated explosive growth even though North Dakota never made the top six list of largest case load. North Dakota had the highest rate in the United States in terms of speed of new infections all 7 weeks. The pandemic accelerated 6 of the 7 weeks. The jerk was positive 3 of the 7 weeks. North Dakota had the highest persistence rate all 7 weeks, meaning new cases today were statistically attributable to novel infections a week ago. There was some underlying condition or events that have echoed forward with increasing numbers of new infections each week. During the week of November 12, 2020, North Dakota had the highest speed of infections in the United States at 175 new infections per 100,000 people; measures of acceleration indicated the speed of infections was increasing at 4.9 new daily infections per 100,000 people per day, which over a week cumulates to over 34 more new daily infections per 100,000 people than a week ago. The jerk of 6.5 new infections per 100,000 people indicates that the state moved from a deceleration the prior week to a rapid acceleration the week of November 12. Finally, North Dakota had the highest persistence effect in the United States at an average of 195.3 new infections per day for the week of November 12 that were statistically attributable to infections the prior week. Not only was North Dakota experiencing explosive growth, the surveillance indicators suggested that the outbreak is still strengthening. Corrective action is needed immediately.

South Dakota has 24 positive upward trending data points for speed, acceleration, jerk, and persistence week over week for 7 weeks, indicating explosive growth. South Dakota had the second highest rate of novel infections and persistence in the United States; for 5 of the 7 weeks, North and South Dakota’s acceleration and jerk were positive, indicating that the pandemic was strengthening at an increasing rate. Utah has 12 data points in Table 2 of the leading six states in speed, acceleration, jerk, and persistence. During the past week of November 12, 2020, Utah had positive acceleration and jerk. Montana had 17 data points, indicating explosive growth. Montana was a top-six state in speed for 6 of the last 7 weeks, acceleration 3 of the last 7 weeks, and jerk 2 of the last 7 weeks. Utah ranked in the top six states for persistence for 6 of the last 7 weeks.

Arizona is more promising. Although Arizona ranked sixth in the rate of new infections during the week of October 1, 2020, and had a leading persistence rate for the weeks of October 1 and October 8, Arizona does not appear in the leading states in Table 2 afterward. Iowa had six indicators and Idaho and Nebraska each had seven indicators of explosive growth.

Wisconsin had a high caseload, and it was in the top six states for speed, acceleration, and persistence. Wisconsin remained a leader in speed across all 7 weeks and cumulatively had the highest number of new infections per 100,000 people over those weeks. Wisconsin’s pandemic accelerated in 3 of the 7 weeks. During the week of October 1, 2020, Wisconsin had a speed of 42.7, and by the week of November 12, 2020, its speed had reached 112.9. Wisconsin ranked as a leading state all 7 weeks for persistence, indicating there was some underlying condition that persisted and echoed forward.

Wyoming showed the largest acceleration over the 7 weeks, with speed accelerating from 19.2 new daily cases per 100,000 people during the week of October 1, 2020, to 125.1 during the week of November 12, 2020.

Table 2 demonstrates the utility of dynamic surveillance metrics because traditional surveillance metrics only report caseloads of each state. Larger states (Table 3) will more likely be captured in those types of metrics, missing the dynamics of the pandemic and missing those states who demonstrate high rates of infection speed, speeds that are accelerating, accelerating speeds that jerk upwards, and those new cases today that are statistically attributable to new cases last week or those new cases with an underlying biological or social condition (eg, attending mass events without face masks or social distancing), that is, echoing forward and leading to additional infections.

**Table 3:** Most populous US states.

State	Population as of 2020
California	39,937,500
Texas	29,472,300
Florida	21,993,000
New York	19,440,500
Pennsylvania	12,820,900
Illinois	12,700,381

**Figure 2:**
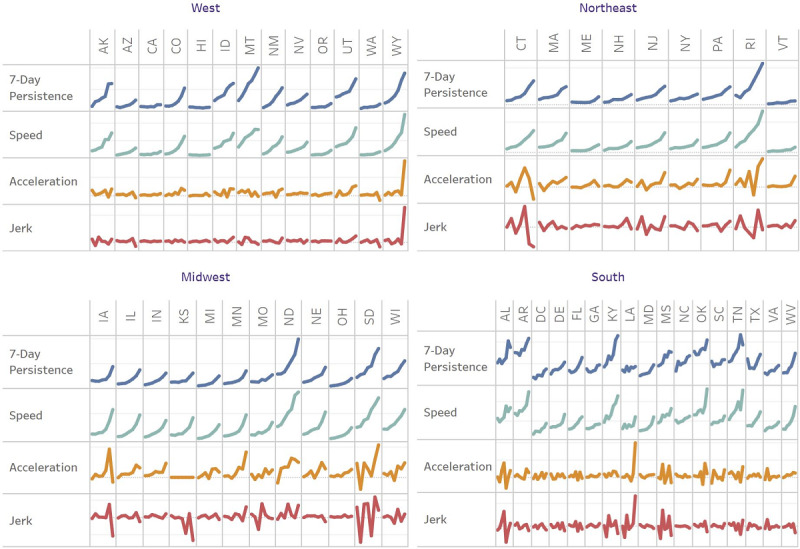
Weekly US state statistics.

Table 4 provides the average of the US states or the US rates for speed, jerk, and 7-day persistence. During the week of October 1, 2020, the speed was positive at 12.91 infections per 100,000 people. In the speed column, for each subsequent week in the study, the speed increased to a maximum speed of 39.38 during the week of November 11, 2020. This explains why the acceleration rate per 100,000 people increased weekly, as acceleration was the measure of increases in speed week over week. In the final column, we see the 7-day persistence begins at 13.99 per 100,000 people during the week of October 1 that are statistically attributed to the number of novel infections 7 days earlier. By the week of November 12, this persistence had increased to 33.62, meaning that 85% of the 39.38 average daily new cases the week of November 12 were statistically attributable to new cases in the prior week. This could happen if, for example, in-restaurant diners last Saturday became infected and then dined in-restaurant again this Saturday and infected other diners, who then eat in-restaurant next Saturday and infect the next cohort, etc. Sequential sports, election, and postelection watch parties could generate the same effect. The novel measures, thus, suggest that a return to stricter guidelines is appropriate for regaining control of the pandemic and were indicated as early as the week of October 22, 2020, which was the third consecutive week of positive acceleration and jerk, and increasing persistence. Weekly trends by state are displayed in Figure 2. See Multimedia Appendices 2-5 for US maps displaying weekly acceleration, jerk, and persistence.

**Table 4:** US pandemic dynamics.

Date	Speed	Acceleration	Jerk	7-day persistence effect
Oct 1, 2020	12.91	0.12	–0.12	13.99
Oct 8, 2020	13.85	0.41	0.13	14.05
Oct 15, 2020	16.03	0.34	0.11	15.08
Oct 22, 2020	18.39	0.45	0.24	17.45
Oct 29, 2020	22.97	0.63	–0.16	20.03
Nov 5, 2020	28.09	1.24	0.18	27.11
Nov 12, 2020	39.38	1.45	–0.34	33.62

## Discussion

### Principal Results

The United States has had an uncoordinated, decentralized response to COVID-19. Although traditional public health surveillance provides a static view of the pandemic, the data are limited by secondary bias from undercounting, reporting delays, testing issues, and other forms of contamination. The novel measures presented in this study take steps toward resolving these shortcomings and measure the dynamics of the pandemic. They also provide greater insight into the evolution of a pandemic, such as where COVID-19 is transmitted and whether rates of transmission are increasing. Measures like 7-day persistence control for incomplete case ascertainment and look retrospectively to understand current infection rates, and speed, acceleration, and jerk provide a dynamic perspective on future cases.

The data presented in Tables S5-S18 in Multimedia Appendix 1 indicate that some states are responding well to the pandemic, while others are responding poorly in the present or suffering from past mistakes. States can be examined systematically to determine their relative performance. At a high level, the static metrics demonstrate national and state burden of disease over a 7-week period. Acceleration and jerk indicate the potential progress of disease burden. A state with a high number of new cases but a negative acceleration and jerk is more likely to have a good trajectory than a state with a high number of cases and a positive acceleration and jerk. The 7-day persistence metric hints at both past decision making and future progress. A state with a high 7-day persistence may have experienced a super-spreader, causing an echo in transmissions to the present and potentially into the future. These echoes may be particularly susceptible to control by identifying the causal event and performing rigorous contact tracing or eliminating the offending behaviors (eg, gatherings without face masks and social distancing).

Overall, the state of the pandemic in the United States is concerning based on static surveillance and dynamic metrics. All 50 states and the District of Colombia have positive rates of new infections. Moreover, the rates of infection are higher and faster than at any other time since the onset of the pandemic.

### Why the COVID-19 Response Differs From Previous Infectious Disease Outbreaks?

COVID-19 has been politicized and polarized, flamed by social media and misinformation [47-56]. Scientists have consistently emphasized that a reduction in COVID-19 transmission is predicated on social distancing, quarantines, hand hygiene, crowd control, and wearing masks [14,16,17]. Others weighed COVID-19 safety measures against shutting down the economy to prevent financial hardships that dynamic surveillance can inform. In the absence of policy actions, COVID-19 experienced exponential growth rates of approximately 38% per day [57]. Countries with anticontagion policies significantly and substantially slowed the growth of COVID-19 [57], yet many US states are unable to implement COVID-19 control policies. Finally, since the start of the COVID-19 pandemic, there was confusion about who is “in charge” of the US epidemic response [58]. Federal government powers do not allow it to impose specific restrictions on states [59]; however, its resources are *vast*, with the ability to develop national guidelines, promote scientific research and vaccine development, and direct resources toward COVID-19 relief [60-62]. Unfortunately, city mayors and state governors must patch together uncoordinated piecemeal COVID-19 control policies [63-65].

### Conflicts in Adopting Policy

The US COVID-19 epidemic has rebounded and is accelerating rapidly with multiple outbreaks in most US states, indicating shortfalls in preparedness and a dearth of nonpharmaceutical control measures. Although standard surveillance techniques such as daily and cumulative infections and deaths are helpful, they have incomplete case ascertainment, err on the side of the most severe cases, and provide a static view of what has already occurred during the pandemic, which is less helpful in prevention. Public health policy that is informed by dynamic surveillance can shift the country from reacting to COVID-19 transmissions to being proactive and taking corrective action when indicators of speed, acceleration, jerk, and persistence remain positive week over week. Implicit within our surveillance is an early warning system that indicates when there is problematic growth in COVID-19 transmissions as well as to signal when growth will become explosive without action.

A public health approach that focuses on prevention can prevent major outbreaks in addition to endorsing effective public health policies and may resolve conflict between various government entities. Moreover, subnational analyses on the dynamics of the pandemic allows us to zero in on where transmissions are increasing, meaning corrective action can be applied with precision in problematic areas. Dynamic public health surveillance can inform specific geographies where quarantines are necessary, preserving the economy in other US areas.

Without a unified national policy to address COVID-19, individual cities and states have launched efforts to control the spread of the pandemic. Similar studies have found that states that imposed strict guidelines saw drastic reductions in COVID-19 spread and avoided significant increased case counts and fatalities [66,67].

Discord between New York City Mayor Bill De Blasio and New York Governor Andrew Cuomo has resulted in delays in shutting down new virus hot spots in neighborhoods across New York City [68]. In other states, governors have faced problems passing public health safety measures with proposed legislation ruled unconstitutional by state courts. In early May, the Wisconsin Supreme Court overturned a statewide stay-at-home order put in place by Governor Evers to control rising cases [69]. Wisconsin has since faced a large increase in COVID-19 cases and reports some of the largest numbers of new infections per day. Recently, Governor Whitmer of Michigan also experienced issues with an executive order she passed when the Michigan Supreme Court ruled her statewide mask mandate as unconstitutional [70]. Even within states, some cities have imposed mask mandates on their own accord, while others have refused, leading to different outcomes. In South Carolina, areas with mask mandates reported a 46% greater reduction in COVID-19 case rates than those areas without mask mandates [71]. The Tri-State area of New Jersey, New York, and Connecticut coordinated travel restrictions, public health guidelines, and economic activities [72]. After initial success, four additional states joined to quell the COVID-19 pandemic [73]. These coordination challenges and frictions between levels of government have been observed in other countries as well, including Brazil, India, and Nigeria [74], and can be especially pronounced in settings of vertically divided authority where subnational governments are led by opposition parties. Germany is a notable exception; states have control over their own health systems and each state was able to pursue its own testing without waiting for approval from a national health laboratory—a factor that has been partially credited for the country’s swift testing response [75].

### Current State of the Pandemic

An important change in the dynamic of COVID-19 transmission occurred starting mid-August when universities and schools around the country started to open for their new academic year [30]. Approximately 53 million US children between the ages of 5-17 years resumed school in fall of 2020 [76]. The CDC confirmed over 2800 COVID-19 cases at the University of Wisconsin within the first month of reopening [77]. Similarly, the University of Missouri has confirmed over 1600 COVID-19 cases among its students since reopening on August 19, 2020 [78]. Reinstating college sports also resulted in novel infections [79].

States such as Wisconsin and Indiana have reported pandemic highs in daily COVID-19 new infection counts [3,80]. As October 2020 began, Utah reported the nation’s highest positivity rate [81]. This trend was high throughout September, suggesting a potential dearth in testing that caused an undercount [81]. Florida, which saw a surge increase over the summer, has seen a steady decline in its daily case counts after stricter measures were put in place by the governor; however, these measures have since been lifted [82]. Wisconsin, Iowa, and Utah have not instituted mask mandates and have seen worsening infection counts that have not improved. The four states of greatest concern are Wisconsin, North Dakota, South Dakota, and Illinois. Wisconsin, North Dakota, and South Dakota have multiple data points indicating explosive growth. Wisconsin and Illinois both top the nation in daily new infections and have positive growth rates. California and Pennsylvania have positive speed, acceleration, jerk, and persistence, suggesting the outbreak in these two populous states will be significant in terms of caseload.

### Conclusion

The variation in speed, acceleration or deceleration, and jerk between and within states is consistent with varying degrees of compliance with public health guidelines to combat COVID-19 in terms of social distancing, masks, hand hygiene, and crowd control. Some states have overall higher caseloads because they have higher populations, such as California, New York, Illinois, Texas, and Florida, and others have alarming speed, acceleration, and jerk. Nationally, the US COVID-19 epidemic has rebounded and is accelerating rapidly in multiple states, indicating shortfalls in preparedness and a dearth of nonpharmaceutical control measures. Although standard surveillance techniques such as daily and cumulative infections and deaths are helpful, they also have incomplete case ascertainment, err on the side of the most severe cases, and provide a static view of what has already occurred in the pandemic, which is less helpful for future planning. Public health policy that is informed by dynamic surveillance can shift the country from reacting to COVID-19 infections and deaths to being proactive and taking corrective action when indicators of speed, acceleration, jerk, and persistence remain positive week over week.

Implicit within our surveillance is an early warning system that indicates when there is problematic growth in COVID-19 transmissions as well as signals when growth is likely. For example, at the US level, the novel metrics indicated a need for strengthening public health measures as early as the week of October 22, 2020, when the highest numbers of new daily cases were 82,000-84,000 per day. In the absence of these novel metrics, little action was taken until the number of new cases had more than doubled to over 161,934 new cases on November 17, 2020 [5]. At the state level, the indicators suggested that explosive growth occurring in the Dakotas, Wisconsin, and Wyoming could also have been identified in late October. Public health actions taken at that time in those and other states and areas might have significantly reduced the number of new cases and prevented the severe overtaxing of hospital and medical resources that is now happening.

A public health approach that focuses on prevention can prevent major outbreaks in addition to confirming when public health guidelines are effective and controlling the pandemic. Moreover, subnational analyses on the dynamics of the pandemic allows us to zero in on where transmissions are increasing, meaning corrective action can be applied with precision on problematic areas. However, this approach requires subnational, dynamic public health surveillance that can inform specific geographies where lockdowns or other measures are necessary. This paper provides novel surveillance measures that can help fill that exact need.

### Limitations

Our data are limited by state-level granularity and differences in testing and reporting within states. State-level granularity, although superior to national reporting, provides less detail than county- or city-level reporting. This limitation is particularly pronounced in states with large urban centers governed by powerful mayors. Testing and reporting varies across and within states for many reasons, including the decentralization of US health care. Both insurers and providers have inconsistent policies in place and resources deployed for COVID-19 testing. To address this need for small area surveillance, we have generated static and dynamic surveillance for larger metropolitan areas in the United States in an additional publication.

### Comparison With Prior Work

This study is part of a broader research program at Northwestern Feinberg School of Medicine, The Global SARS-CoV-2 Surveillance Project: Policy, Persistence, & Transmission. This research program developed novel surveillance metrics to include rates of speed, acceleration, jerk, and 7-day persistence [28,30]. We have also derived surveillance metrics for all global regions.

## Appendix

Multimedia Appendix 1 Supplementary Tables S5-S18.

Multimedia Appendix 2 Explosive growth potential.

Multimedia Appendix 3 US weekly SARS-CoV-2 trends.

Multimedia Appendix 4 Weekly US 7-day persistence map.

Multimedia Appendix 5 Weekly US acceleration and jerk.
