# Supplementary material for: Dynamic Public Health Surveillance to Track and Mitigate the US COVID-19 Epidemic: Longitudinal Trend Analysis Study
Source: J Med Internet Res. 2020 Dec 3;22(12):e24286. doi: 10.2196/24286 (PMC7717896; doi:10.2196/24286)
Supplement: Multimedia Appendix 1 [file jmir_v22i12e24286_app1.docx]

**Table S5. Static Surveillance Metrics for the Week of 9/28-10/4**

| **State** | **Cumulative COVID Cases** | **7 Day Moving Average New Cases** | **Infection Rate per 100k** | **Cumulative Deaths** | **7 Day Moving Average of Deaths** |
| --- | --- | --- | --- | --- | --- |
| AK | 8912 | 138.71 | 18.0 | 57 | 1.71 |
| AL | 155744 | 1076.86 | 21.3 | 2548 | 6 |
| AR | 84821 | 824.57 | 37.2 | 1384 | 19.71 |
| AZ | 219212 | 480.00 | 9.7 | 5674 | 16.43 |
| CA | 813687 | 3292.43 | 7.7 | 15888 | 82 |
| CO | 70536 | 552.43 | 8.9 | 1960 | 4.86 |
| CT | 57742 | 181.43 | 5.4 | 4511 | 1.71 |
| DC | 15358 | 36.00 | 4.5 | 628 | 1 |
| DE | 20787 | 120.00 | 17.9 | 636 | 0.86 |
| FL | 700602 | 2250.71 | 11.9 | 14619 | 94 |
| GA | 319334 | 1184.00 | 12.3 | 7063 | 34.43 |
| HI | 12589 | 105.86 | 8.5 | 136 | 2 |
| IA | 86634 | 794.71 | 32.9 | 1360 | 8.29 |
| ID | 42048 | 472.14 | 34.4 | 469 | 2.14 |
| IL | 297929 | 2051.43 | 17.1 | 8940 | 23.71 |
| IN | 121176 | 991.43 | 17.2 | 3645 | 13.86 |
| KS | 59749 | 646.14 | 0.0 | 678 | 8.14 |
| KY | 69728 | 795.71 | 19.9 | 1191 | 7.71 |
| LA | 168009 | 509.00 | 11.9 | 5519 | 13.71 |
| MA | 132870 | 553.86 | 10.9 | 9480 | 16.86 |
| MD | 125510 | 530.00 | 13.0 | 3949 | 5.71 |
| ME | 5431 | 30.86 | 3.0 | 142 | 0.29 |
| MI | 139012 | 953.57 | 10.0 | 7102 | 11.86 |
| MN | 100200 | 1026.86 | 18.9 | 2102 | 8.86 |
| MO | 127912 | 1371.57 | 29.3 | 2128 | 25.14 |
| MS | 98886 | 510.86 | 23.4 | 2979 | 15 |
| MT | 13500 | 322.57 | 40.1 | 181 | 2.29 |
| NC | 212909 | 2102.86 | 21.7 | 3579 | 31.86 |
| ND | 22218 | 395.29 | 48.8 | 191 | 5 |
| NE | 45564 | 469.43 | 26.9 | 478 | 2.29 |
| NH | 8317 | 39.00 | 3.8 | 441 | 0.43 |
| NJ | 205889 | 619.57 | 6.9 | 16127 | 5.71 |
| NM | 29435 | 206.86 | 13.3 | 877 | 2.86 |
| NV | 80410 | 459.00 | 14.0 | 1603 | 5.57 |
| NY | 460031 | 1026.29 | 7.1 | 25490 | 7.29 |
| OH | 155314 | 1081.43 | 11.4 | 4817 | 14.57 |
| OK | 88369 | 1017.86 | 29.6 | 1035 | 7.71 |
| OR | 33862 | 285.29 | 8.4 | 560 | 3 |
| PA | 160123 | 960.86 | 9.0 | 8160 | 11.57 |
| RI | 7182943 | 34607.00 | 13.8 | 199040 | 696.86 |
| SC | 24914 | 86.14 | 15.7 | 1117 | 1.57 |
| SD | 148323 | 802.29 | 7.4 | 3400 | 17.29 |
| TN | 23136 | 434.14 | 84.4 | 236 | 3.71 |
| TX | 197432 | 1412.57 | 18.9 | 2501 | 27.29 |
| UT | 752501 | 4083.14 | 12.2 | 15823 | 79.43 |
| VA | 74050 | 990.14 | 31.4 | 459 | 2.14 |
| VT | 148721 | 747.00 | 5.3 | 3228 | 16.43 |
| WA | 1755 | 4.43 | 0.5 | 58 | 0 |
| WI | 87522 | 545.71 | 6.3 | 2126 | 6.43 |
| WV | 132123 | 2489.00 | 51.5 | 1358 | 11.86 |
| WY | 16024 | 188.29 | 9.8 | 354 | 4.14 |
| Region | 6083 | 111.14 | 23.3 | 53 | 0.43 |

**Table S6. Static Surveillance Metrics for the Week of 10/5-10/11**

| **State** | **Cumulative COVID Cases** | **7 Day Moving Average of New Cases** | **Infection Rate per 100k** | **Cumulative Deaths** | **7 Day Moving Average of Deaths** |
| --- | --- | --- | --- | --- | --- |
| AK | 9996 | 154.9 | 18.5 | 60 | 0.4 |
| AL | 161975 | 890.1 | 11.4 | 2637 | 12.7 |
| AR | 90145 | 760.6 | 41.9 | 1503 | 17.0 |
| AZ | 223401 | 598.4 | 11.9 | 5743 | 9.9 |
| CA | 834800 | 3016.1 | 9.0 | 16361 | 67.6 |
| CO | 74922 | 626.6 | 12.7 | 1990 | 4.3 |
| CT | 59748 | 286.6 | 10.8 | 4527 | 2.3 |
| DC | 15765 | 58.1 | 9.6 | 634 | 0.9 |
| DE | 21682 | 127.9 | 13.6 | 651 | 2.1 |
| FL | 717148 | 2363.7 | 15.1 | 15254 | 90.7 |
| GA | 327407 | 1153.3 | 11.9 | 7294 | 33.0 |
| HI | 13232 | 91.9 | 7.6 | 163 | 3.9 |
| IA | 92145 | 787.3 | 40.0 | 1421 | 8.7 |
| ID | 45753 | 529.3 | 37.5 | 500 | 4.4 |
| IL | 313439 | 2215.7 | 24.1 | 9159 | 31.3 |
| IN | 129677 | 1214.4 | 21.5 | 3742 | 13.9 |
| KS | 63952 | 600.4 | 0.0 | 723 | 6.4 |
| KY | 77455 | 1103.9 | 19.4 | 1234 | 6.1 |
| LA | 173149 | 734.3 | 11.3 | 5609 | 12.9 |
| MA | 136936 | 580.9 | 6.4 | 9565 | 12.1 |
| MD | 129425 | 559.3 | 12.6 | 3979 | 4.3 |
| ME | 5639 | 29.7 | 2.6 | 142 | 0.0 |
| MI | 146493 | 1068.7 | 14.0 | 7193 | 13.0 |
| MN | 107922 | 1103.1 | 22.5 | 2160 | 8.3 |
| MO | 137156 | 1320.6 | 24.5 | 2259 | 18.7 |
| MS | 102819 | 561.9 | 19.4 | 3074 | 13.6 |
| MT | 16677 | 453.9 | 57.4 | 197 | 2.3 |
| NC | 225397 | 1784.0 | 23.2 | 3722 | 20.4 |
| ND | 25384 | 452.3 | 69.2 | 234 | 6.1 |
| NE | 49396 | 547.4 | 33.0 | 507 | 4.1 |
| NH | 8878 | 80.1 | 5.7 | 449 | 1.1 |
| NJ | 211148 | 751.3 | 14.6 | 16160 | 4.7 |
| NM | 31372 | 276.7 | 20.3 | 896 | 2.7 |
| NV | 83827 | 488.1 | 15.6 | 1649 | 6.6 |
| NY | 470104 | 1439.0 | 9.4 | 25555 | 9.3 |
| OH | 164262 | 1278.3 | 13.2 | 4983 | 23.7 |
| OK | 95564 | 1027.9 | 30.6 | 1085 | 7.1 |
| OR | 36116 | 322.0 | 11.4 | 594 | 4.9 |
| PA | 167928 | 1115.0 | 10.7 | 8299 | 19.9 |
| RI | 26045 | 161.6 | 25.4 | 1127 | 1.4 |
| SC | 154755 | 918.9 | 20.4 | 3514 | 16.3 |
| SD | 26411 | 467.9 | 57.1 | 272 | 5.1 |
| TN | 209447 | 1716.4 | 29.2 | 2705 | 29.1 |
| TX | 781794 | 4184.7 | 14.6 | 16334 | 73.0 |
| UT | 81947 | 1128.1 | 46.8 | 501 | 6.0 |
| VA | 155535 | 973.4 | 21.6 | 3328 | 14.3 |
| VT | 1838 | 11.9 | 1.8 | 58 | 0.0 |
| WA | 91208 | 526.6 | 7.2 | 2177 | 7.3 |
| WI | 149467 | 2477.7 | 56.2 | 1435 | 11.0 |
| WV | 17325 | 185.9 | 10.4 | 370 | 2.3 |
| WY | 7092 | 144.1 | 33.3 | 54 | 0.1 |
| Region | 7501098 | 38507.9 | 16.7 | 203782 | 677.4 |

**Table S7. Static Surveillance Metrics for the Week of 10/12-10/18**

| **State** | **Cumulative COVID Cases** | **7 Day Moving Average New Cases** | **Infection Rate per 100k** | **Cumulative Deaths** | **7 Day Moving Average of Deaths** |
| --- | --- | --- | --- | --- | --- |
| AK | 11348 | 193.14 | 22.56 | 65 | 0.7 |
| AL | 169162 | 1026.71 | 24.17 | 2756 | 17 |
| AR | 96524 | 911.29 | 42.35 | 1645 | 20.3 |
| AZ | 228748 | 763.86 | 15.29 | 5789 | 6.6 |
| CA | 858401 | 3371.57 | 8.43 | 16757 | 56.6 |
| CO | 80777 | 836.43 | 12.02 | 2025 | 5 |
| CT | 62028 | 325.71 | 4.68 | 4540 | 1.9 |
| DC | 16166 | 57.29 | 4.82 | 638 | 0.6 |
| DE | 22560 | 125.43 | 9.76 | 661 | 1.4 |
| FL | 735685 | 2648.14 | 15.30 | 15932 | 96.9 |
| GA | 336241 | 1262.00 | 15.45 | 7492 | 28.3 |
| HI | 13872 | 91.43 | 6.99 | 183 | 2.9 |
| IA | 98717 | 938.86 | 42.25 | 1506 | 12.1 |
| ID | 49892 | 591.29 | 36.09 | 516 | 2.3 |
| IL | 334658 | 3031.29 | 31.68 | 9373 | 30.6 |
| IN | 141212 | 1647.86 | 28.86 | 3864 | 17.4 |
| KS | 69155 | 743.29 | 0.00 | 838 | 16.4 |
| KY | 84195 | 962.86 | 26.46 | 1296 | 8.9 |
| LA | 176952 | 543.29 | 15.98 | 5707 | 14 |
| MA | 141579 | 663.29 | 8.52 | 9672 | 15.3 |
| MD | 133548 | 589.00 | 10.42 | 4028 | 7 |
| ME | 5836 | 28.14 | 1.49 | 144 | 0.3 |
| MI | 156913 | 1488.57 | 24.61 | 7302 | 15.6 |
| MN | 117106 | 1312.00 | 20.62 | 2199 | 5.6 |
| MO | 150554 | 1914.00 | 30.55 | 2442 | 26.1 |
| MS | 108139 | 760.00 | 44.42 | 3152 | 11.1 |
| MT | 20933 | 608.00 | 67.65 | 230 | 4.7 |
| NC | 238939 | 1934.57 | 24.14 | 3874 | 21.7 |
| ND | 29653 | 609.86 | 92.64 | 289 | 7.9 |
| NE | 54467 | 724.43 | 47.77 | 530 | 3.3 |
| NH | 9426 | 78.29 | 5.66 | 463 | 2 |
| NJ | 216994 | 835.14 | 10.93 | 16197 | 5.3 |
| NM | 34290 | 416.86 | 27.52 | 921 | 3.6 |
| NV | 87969 | 591.71 | 21.27 | 1698 | 7.0 |
| NY | 479400 | 1328.00 | 7.51 | 25618 | 9.0 |
| OH | 175843 | 1654.43 | 18.63 | 5038 | 7.9 |
| OK | 103836 | 1181.71 | 30.86 | 1143 | 8.3 |
| OR | 38525 | 344.14 | 8.65 | 611 | 2.4 |
| PA | 177520 | 1370.29 | 12.48 | 8432 | 19 |
| RI | 27438 | 199.00 | 25.86 | 1149 | 3.1 |
| SC | 161106 | 907.29 | 25.19 | 3607 | 13.3 |
| SD | 31012 | 657.29 | 90.09 | 304 | 4.6 |
| TN | 222827 | 1911.43 | 33.52 | 2864 | 22.7 |
| TX | 809808 | 4002.00 | 16.30 | 16812 | 68.3 |
| UT | 90461 | 1216.29 | 46.73 | 529 | 4 |
| VA | 162941 | 1058.00 | 15.59 | 3388 | 8.6 |
| VT | 1903 | 9.29 | 2.24 | 58 | 0 |
| WA | 95509 | 614.43 | 9.64 | 2221 | 6.3 |
| WI | 171122 | 3093.57 | 69.39 | 1565 | 18.6 |
| WV | 19082 | 251.00 | 14.73 | 393 | 3.3 |
| WY | 8375 | 183.29 | 34.21 | 57 | 0.4 |
| Region | 7869347 | 44591.71 | 19.03 | 208513 | 675.9 |

**Table S8. Static Surveillance Metrics for the Week of 10/19-10/25**

| **State** | **Cumulative COVID Cases** | **7 Day Moving Average New Cases** | **Infection Rate per 100k** | **Cumulative Deaths** | **7 Day Moving Average of Deaths** |
| --- | --- | --- | --- | --- | --- |
| AK | 12877 | 218 | 31.85 | 68 | 0.43 |
| AL | 177064 | 1129 | 51.72 | 2843 | 12.43 |
| AR | 102798 | 896 | 39.83 | 1772 | 18.14 |
| AZ | 234906 | 880 | 13.66 | 5859 | 10 |
| CA | 880724 | 3189 | 7.44 | 17189 | 61.71 |
| CO | 88849 | 1153 | 22.00 | 2066 | 5.86 |
| CT | 65373 | 478 | 14.08 | 4569 | 4.14 |
| DC | 16537 | 53 | 5.53 | 642 | 0.57 |
| DE | 23528 | 138 | 15.71 | 670 | 1.29 |
| FL | 758306 | 3232 | 25.43 | 16470 | 76.86 |
| GA | 345535 | 1328 | 16.81 | 7729 | 33.86 |
| HI | 14427 | 79 | 5.44 | 203 | 2.86 |
| IA | 105625 | 987 | 45.48 | 1601 | 13.57 |
| ID | 55650 | 823 | 55.23 | 546 | 4.29 |
| IL | 363746 | 4155 | 39.00 | 9647 | 39.14 |
| IN | 155246 | 2005 | 42.33 | 4065 | 28.71 |
| KS | 74456 | 757 | 0.00 | 952 | 16.29 |
| KY | 92299 | 1158 | 29.17 | 1380 | 12.00 |
| LA | 181904 | 707 | 16.61 | 5799 | 13.14 |
| MA | 147215 | 805 | 15.22 | 9810 | 19.71 |
| MD | 137979 | 633 | 12.29 | 4070 | 6 |
| ME | 6064 | 33 | 2.75 | 146 | 0.29 |
| MI | 170076 | 1880 | 22.07 | 7464 | 23.14 |
| MN | 128152 | 1578 | 27.68 | 2301 | 14.57 |
| MO | 162723 | 1738 | 30.21 | 2657 | 30.71 |
| MS | 113081 | 706 | 32.19 | 3231 | 11.29 |
| MT | 25640 | 672 | 86.83 | 278 | 6.86 |
| NC | 252992 | 2008 | 22.88 | 4082 | 29.71 |
| ND | 35052 | 771 | 116.39 | 338 | 7 |
| NE | 60308 | 834 | 46.47 | 576 | 6.57 |
| NH | 9994 | 81 | 5.66 | 470 | 1.00 |
| NJ | 224385 | 1056 | 13.08 | 16263 | 9.43 |
| NM | 38715 | 632 | 39.06 | 950 | 4.14 |
| NV | 92853 | 698 | 25.62 | 1736 | 5.43 |
| NY | 490134 | 1533 | 8.37 | 25694 | 10.86 |
| OH | 190430 | 2084 | 20.75 | 5161 | 17.57 |
| OK | 112483 | 1235 | 41.14 | 1221 | 11.14 |
| OR | 40810 | 326 | 8.70 | 646 | 5 |
| PA | 188360 | 1549 | 16.11 | 8592 | 22.86 |
| RI | 29594 | 308 | 44.46 | 1173 | 3.43 |
| SC | 167485 | 911 | 21.91 | 3755 | 21.14 |
| SD | 36017 | 715 | 109.99 | 347 | 6.14 |
| TN | 237907 | 2154 | 29.96 | 3011 | 21.00 |
| TX | 845100 | 5042 | 21.70 | 17286 | 67.71 |
| UT | 99549 | 1298 | 48.13 | 563 | 4.86 |
| VA | 170104 | 1023 | 15.61 | 3524 | 19.43 |
| VT | 1987 | 12 | 2.56 | 58 | 0 |
| WA | 99874 | 624 | 9.51 | 2286 | 9.29 |
| WI | 195823 | 3529 | 62.38 | 1725 | 22.86 |
| WV | 21057 | 282 | 18.02 | 418 | 3.57 |
| WY | 10119 | 249 | 46.82 | 68 | 1.57 |
| Region | 8291912 | 50751 | 22.15 | 213970 | 779.57 |

**Table S9. Static Surveillance Metrics for the Week of 10/26-11/1**

| **State** | **Cumulative COVID Cases** | **7 Day Moving Average New Cases** | **Infection Rate per 100k** | **Cumulative Deaths** | **7 Day Moving Average of Deaths** |
| --- | --- | --- | --- | --- | --- |
| AK | 15522 | 377.86 | 50.17 | 77 | 1.29 |
| AL | 189149 | 1726.43 | 29.43 | 2914 | 10.14 |
| AR | 109712 | 987.71 | 35.52 | 1894 | 17.43 |
| AZ | 242480 | 1082.00 | 18.07 | 5918 | 8.43 |
| CA | 912904 | 4597.14 | 10.61 | 17541 | 50.29 |
| CO | 100208 | 1622.71 | 25.61 | 2094 | 4.00 |
| CT | 70446 | 724.71 | 37.00 | 4609 | 5.71 |
| DC | 17074 | 76.71 | 14.31 | 645 | 0.43 |
| DE | 24553 | 146.43 | 16.53 | 689 | 2.71 |
| FL | 784331 | 3717.86 | 19.14 | 16854 | 54.86 |
| GA | 356848 | 1616.14 | 17.17 | 7923 | 27.71 |
| HI | 15039 | 87.43 | 4.31 | 213 | 1.43 |
| IA | 114982 | 1336.71 | 61.49 | 1693 | 13.14 |
| ID | 61785 | 876.43 | 48.24 | 599 | 7.57 |
| IL | 400171 | 5203.57 | 50.21 | 9945 | 42.57 |
| IN | 172730 | 2497.71 | 53.74 | 4260 | 27.86 |
| KS | 82045 | 1084.14 | 0.00 | 1007 | 7.86 |
| KY | 103305 | 1572.29 | 40.54 | 1461 | 11.57 |
| LA | 186216 | 616.00 | 8.48 | 5908 | 15.57 |
| MA | 155564 | 1192.71 | 19.53 | 9951 | 20.14 |
| MD | 143387 | 772.57 | 15.91 | 4127 | 8.14 |
| ME | 6467 | 57.57 | 5.95 | 146 | 0.00 |
| MI | 190043 | 2852.43 | 41.14 | 7653 | 27.00 |
| MN | 142311 | 2022.71 | 50.84 | 2419 | 16.86 |
| MO | 177693 | 2138.57 | 49.87 | 2899 | 34.57 |
| MS | 118587 | 786.57 | 32.59 | 3310 | 11.29 |
| MT | 30853 | 744.71 | 82.99 | 337 | 8.43 |
| NC | 269021 | 2289.86 | 27.51 | 4283 | 28.71 |
| ND | 41130 | 868.29 | 160.49 | 382 | 6.29 |
| NE | 66545 | 891.00 | 60.43 | 628 | 7.43 |
| NH | 10768 | 110.57 | 9.34 | 482 | 1.71 |
| NJ | 234547 | 1451.71 | 17.45 | 16332 | 9.86 |
| NM | 43826 | 730.14 | 31.33 | 991 | 5.86 |
| NV | 98554 | 814.43 | 34.90 | 1769 | 4.71 |
| NY | 503176 | 1863.14 | 12.85 | 25792 | 14.00 |
| OH | 208937 | 2643.86 | 30.71 | 5275 | 16.29 |
| OK | 120193 | 1101.43 | 26.31 | 1306 | 12.14 |
| OR | 43793 | 426.14 | 13.40 | 673 | 3.86 |
| PA | 202876 | 2073.71 | 17.20 | 8762 | 24.29 |
| RI | 32312 | 388.29 | 34.74 | 1195 | 3.14 |
| SC | 174591 | 1015.14 | 21.36 | 3889 | 19.14 |
| SD | 43000 | 997.57 | 113.04 | 403 | 8.00 |
| TN | 256880 | 2710.43 | 38.95 | 3263 | 36.00 |
| TX | 886820 | 5960.00 | 23.54 | 17819 | 76.14 |
| UT | 110640 | 1584.43 | 57.30 | 598 | 5.00 |
| VA | 178183 | 1154.14 | 16.74 | 3636 | 16.00 |
| VT | 2141 | 22.00 | 3.37 | 58 | 0.00 |
| WA | 104743 | 695.57 | 9.40 | 2353 | 9.57 |
| WI | 226705 | 4411.71 | 88.38 | 2003 | 39.71 |
| WV | 23466 | 344.14 | 22.43 | 443 | 3.57 |
| WY | 12507 | 341.14 | 62.37 | 87 | 2.71 |
| Region | 8819759 | 63893.00 | 26.55 | 219508 | 791.14 |

**Table S10. Static Surveillance Metrics for the Week of 11/2-11/8**

| **State** | **Cumulative COVID Cases** | **7 Day Moving Average New Cases** | **Infection Rate per 100k** | **Cumulative Deaths** | **7 Day Moving Average of Deaths** |
| --- | --- | --- | --- | --- | --- |
| AK | 18174 | 378.86 | 42.92 | 84 | 1 |
| AL | 199158 | 1429.86 | 28.17 | 3026 | 16 |
| AR | 117360 | 1092.57 | 51.30 | 2037 | 20.43 |
| AZ | 252768 | 1469.71 | 29.33 | 6087 | 24.14 |
| CA | 944576 | 4524.57 | 11.56 | 17815 | 39.14 |
| CO | 117637 | 2489.86 | 50.84 | 2147 | 7.57 |
| CT | 77060 | 944.86 | 47.32 | 4656 | 6.71 |
| DC | 17682 | 86.86 | 11.48 | 650 | 0.71 |
| DE | 25753 | 171.43 | 22.49 | 716 | 3.86 |
| FL | 816376 | 4577.86 | 28.49 | 17170 | 45.14 |
| GA | 368368 | 1645.71 | 18.05 | 8578 | 93.57 |
| HI | 15686 | 92.43 | 10.95 | 219 | 0.86 |
| IA | 130732 | 2250.00 | 126.53 | 1802 | 15.57 |
| ID | 68314 | 932.71 | 72.19 | 664 | 9.29 |
| IL | 453750 | 7654.14 | 78.40 | 10313 | 52.57 |
| IN | 196176 | 3349.43 | 65.54 | 4511 | 35.86 |
| KS | 92215 | 1452.86 | 0.00 | 1087 | 11.43 |
| KY | 115277 | 1710.29 | 50.76 | 1534 | 10.43 |
| LA | 190845 | 661.29 | 14.65 | 5995 | 12.43 |
| MA | 165161 | 1371.00 | 27.01 | 10085 | 19.14 |
| MD | 149964 | 939.57 | 19.82 | 4182 | 7.86 |
| ME | 7260 | 113.29 | 13.61 | 150 | 0.57 |
| MI | 218263 | 4031.43 | 61.11 | 7833 | 25.71 |
| MN | 164865 | 3222.00 | 69.90 | 2555 | 19.43 |
| MO | 196576 | 2697.57 | 57.89 | 3106 | 29.57 |
| MS | 123887 | 757.14 | 54.16 | 3405 | 13.57 |
| MT | 36968 | 873.57 | 94.78 | 407 | 10 |
| NC | 285661 | 2377.14 | 27.26 | 4548 | 37.86 |
| ND | 49837 | 1243.86 | 201.56 | 468 | 12.29 |
| NE | 75888 | 1334.71 | 94.50 | 669 | 5.86 |
| NH | 11808 | 148.57 | 18.02 | 486 | 0.57 |
| NJ | 247219 | 1810.29 | 22.09 | 16403 | 10.14 |
| NM | 50251 | 917.86 | 48.22 | 1059 | 9.71 |
| NV | 105360 | 972.29 | 41.13 | 1824 | 7.86 |
| NY | 518812 | 2233.71 | 15.41 | 25892 | 14.29 |
| OH | 235170 | 3747.57 | 42.44 | 5461 | 26.57 |
| OK | 129873 | 1382.86 | 53.10 | 1413 | 15.29 |
| OR | 47839 | 578.00 | 18.73 | 710 | 5.29 |
| PA | 220566 | 2527.14 | 22.65 | 8937 | 25 |
| RI | 35750 | 491.14 | 59.28 | 1222 | 3.86 |
| SC | 181639 | 1006.86 | 14.94 | 3992 | 14.71 |
| SD | 51151 | 1164.43 | 153.73 | 482 | 11.29 |
| TN | 271771 | 2127.29 | 28.83 | 3509 | 35.14 |
| TX | 934994 | 6882.00 | 29.64 | 18453 | 90.57 |
| UT | 124292 | 1950.29 | 87.56 | 632 | 4.86 |
| VA | 187202 | 1288.43 | 16.00 | 3688 | 7.43 |
| VT | 2303 | 23.14 | 5.77 | 58 | 0 |
| WA | 111480 | 962.43 | 19.29 | 2416 | 9 |
| WI | 263571 | 5266.57 | 107.93 | 2269 | 38 |
| WV | 26547 | 440.14 | 31.25 | 480 | 5.29 |
| WY | 15409 | 414.57 | 63.07 | 105 | 2.57 |
| Region | 9465244 | 78565.57 | 35.27 | 225990 | 926 |

**Table S11. Static Surveillance Metrics for the Week of 11/9-11/15**

| **State** | **Cumulative COVID Cases** | **7 Day Moving Average of New Cases** | **Infection Rate per 100k** | **Cumulative Deaths** | **7 Day Moving Average of Deaths** |
| --- | --- | --- | --- | --- | --- |
| AK | 21812 | 519.71 | 65.75 | 96 | 1.71 |
| AL | 210637 | 1639.86 | 40.79 | 3213 | 26.71 |
| AR | 128006 | 1520.86 | 59.94 | 2144 | 15.29 |
| AZ | 266562 | 1970.57 | 19.22 | 6240 | 21.86 |
| CA | 991609 | 6719.00 | 17.53 | 18108 | 41.86 |
| CO | 142402 | 3537.86 | 69.03 | 2201 | 7.71 |
| CT | 85899 | 1262.71 | 32.48 | 4726 | 10 |
| DC | 18507 | 117.86 | 18.14 | 657 | 1 |
| DE | 27546 | 256.14 | 20.95 | 732 | 2.29 |
| FL | 851825 | 5064.14 | 25.63 | 17585 | 59.29 |
| GA | 380190 | 1688.86 | 23.51 | 8881 | 43.29 |
| HI | 16437 | 107.29 | 8.26 | 222 | 0.43 |
| IA | 155603 | 3553.00 | 115.69 | 1930 | 18.29 |
| ID | 77121 | 1258.14 | 94.74 | 733 | 9.86 |
| IL | 536542 | 11827.43 | 100.24 | 10846 | 76.14 |
| IN | 230965 | 4969.86 | 97.90 | 4813 | 43.14 |
| KS | 109225 | 2430.00 | 0.00 | 1215 | 18.29 |
| KY | 129680 | 2057.57 | 52.29 | 1622 | 12.57 |
| LA | 198531 | 1098.00 | 82.37 | 6097 | 14.57 |
| MA | 180189 | 2146.86 | 38.42 | 10242 | 22.43 |
| MD | 159900 | 1419.43 | 24.43 | 4261 | 11.29 |
| ME | 8395 | 162.14 | 14.36 | 159 | 1.29 |
| MI | 259183 | 5845.71 | 73.21 | 8185 | 50.29 |
| MN | 201795 | 5275.71 | 128.11 | 2793 | 34 |
| MO | 225371 | 4113.57 | 75.00 | 3339 | 33.29 |
| MS | 130665 | 968.29 | 42.71 | 3514 | 15.57 |
| MT | 43031 | 866.14 | 89.92 | 472 | 9.29 |
| NC | 303454 | 2541.86 | 27.58 | 4706 | 22.57 |
| ND | 59173 | 1333.71 | 236.20 | 553 | 12.14 |
| NE | 89942 | 2007.71 | 114.20 | 731 | 8.86 |
| NH | 13470 | 237.43 | 23.68 | 495 | 1.29 |
| NJ | 266986 | 2823.86 | 39.30 | 16495 | 13.14 |
| NM | 59034 | 1254.71 | 70.92 | 1158 | 14.14 |
| NV | 114880 | 1360.00 | 47.69 | 1880 | 8 |
| NY | 545762 | 3850.00 | 24.66 | 26055 | 23.29 |
| OH | 274457 | 5612.43 | 60.75 | 5658 | 28.14 |
| OK | 144691 | 2116.86 | 59.57 | 1481 | 9.71 |
| OR | 53879 | 862.86 | 26.29 | 746 | 5.14 |
| PA | 248856 | 4041.43 | 42.87 | 9194 | 36.71 |
| RI | 40764 | 716.29 | 93.26 | 1250 | 4 |
| SC | 190490 | 1264.43 | 29.04 | 4084 | 13.14 |
| SD | 60716 | 1366.43 | 228.34 | 567 | 12.14 |
| TN | 296725 | 3564.86 | 48.97 | 3788 | 39.86 |
| TX | 993841 | 8406.71 | 29.18 | 19147 | 99.14 |
| UT | 143639 | 2763.86 | 122.24 | 687 | 7.86 |
| VA | 198027 | 1546.43 | 17.82 | 3758 | 10 |
| VT | 2651 | 49.71 | 18.59 | 59 | 0.14 |
| WA | 120011 | 1218.71 | 0.00 | 2482 | 9.43 |
| WI | 309572 | 6571.57 | 141.23 | 2621 | 50.29 |
| WV | 30897 | 621.43 | 38.84 | 555 | 10.71 |
| WY | 20479 | 724.29 | 190.93 | 127 | 3.14 |
| Region | 10370024 | 111629.14 | 45.42 | 233303 | 1044.71 |

**Table S12. Novel Surveillance Metrics for the Week of 09/28-10/04**

| **State** | **SPEED: Daily positives per 100K (weekly average of new daily cases per 100K)** | **ACCELERATION: day-to-day change in the number of positives per day, weekly average, per 100K** | **JERK: week over week change in ACCELERATION, per 100K** | **7-DAY PERSISTENCE EFFECT on SPEED**  **(# new cases per day per 100K attributed to new cases 7 days ago)** |
| --- | --- | --- | --- | --- |
| AK | 18.96 | 2.58 | 1.58 | 9.99 |
| AL | 21.96 | -0.03 | -1.71 | 20.46 |
| AR | 27.32 | 0.18 | 0.37 | 30.09 |
| AZ | 6.59 | 0.27 | 0.49 | 8.96 |
| CA | 8.33 | -0.04 | -0.06 | 9.62 |
| CO | 9.59 | -0.26 | 0.03 | 10.76 |
| CT | 5.09 | 0.14 | -0.12 | 4.74 |
| DC | 5.10 | -0.49 | -0.43 | 6.97 |
| DE | 12.32 | 0.84 | 0.65 | 10.05 |
| FL | 10.48 | 0.05 | 0.52 | 13.28 |
| GA | 11.15 | -0.08 | -0.43 | 14.86 |
| HI | 7.48 | -0.47 | -0.72 | 8.44 |
| IA | 25.19 | -0.50 | -0.88 | 29.80 |
| ID | 26.42 | 1.74 | 1.22 | 23.07 |
| IL | 16.19 | -0.10 | -0.58 | 16.29 |
| IN | 14.73 | 0.55 | 0.03 | 12.92 |
| KS | 22.18 | 0.00 | 0.72 | 23.26 |
| KY | 17.81 | 0.79 | 0.16 | 16.67 |
| LA | 10.95 | -0.08 | -0.06 | 13.73 |
| MA | 8.04 | 0.57 | 0.59 | 6.47 |
| MD | 8.77 | 0.67 | 0.60 | 8.44 |
| ME | 2.30 | -0.04 | -0.35 | 2.93 |
| MI | 9.55 | -0.11 | -0.53 | 8.75 |
| MN | 18.21 | 0.39 | 0.37 | 17.35 |
| MO | 22.35 | 1.01 | 1.54 | 25.29 |
| MS | 17.17 | -0.20 | -0.20 | 17.64 |
| MT | 30.18 | 1.32 | -0.48 | 23.22 |
| NC | 20.05 | 0.80 | 0.06 | 12.78 |
| ND | 51.87 | -1.84 | -1.31 | 55.69 |
| NE | 24.27 | 0.20 | -0.31 | 22.99 |
| NH | 2.87 | 0.15 | -0.02 | 2.63 |
| NJ | 6.98 | 0.08 | -0.41 | 5.59 |
| NM | 9.87 | 0.55 | 0.11 | 7.02 |
| NV | 14.90 | 0.19 | 0.76 | 13.14 |
| NY | 5.28 | 0.31 | 0.07 | 4.47 |
| OH | 9.25 | 0.41 | 0.19 | 8.20 |
| OK | 25.72 | 0.31 | 0.71 | 31.16 |
| OR | 6.76 | -0.03 | -0.13 | 7.43 |
| PA | 7.51 | 0.34 | 0.05 | 6.65 |
| RI | 8.13 | 0.43 | -0.36 | 12.09 |
| SC | 15.58 | -1.78 | -0.14 | 21.94 |
| SD | 49.07 | 4.59 | 5.44 | 42.40 |
| TN | 20.68 | 0.96 | 1.03 | 21.42 |
| TX | 14.08 | -0.39 | -1.52 | 24.20 |
| UT | 30.88 | -0.85 | -0.98 | 31.35 |
| VA | 8.75 | -0.76 | -1.05 | 10.99 |
| VT | 0.71 | 0.02 | -0.02 | 0.47 |
| WA | 7.17 | -0.05 | -0.81 | 5.12 |
| WI | 42.75 | 1.28 | -0.10 | 37.74 |
| WV | 10.51 | -0.21 | -0.49 | 11.08 |
| WY | 19.20 | -0.02 | 0.62 | 17.55 |
| Region | 12.91 | 0.12 | -0.12 | 13.99 |

**Table S13. Novel Surveillance Metrics for the Week of 10/5-10/11**

| **State** | **SPEED: Daily positives per 100K (weekly average of new daily cases per 100K)** | **ACCELERATION: day-to-day change in the number of positives per day, weekly average, per 100K** | **JERK: week over week change in ACCELERATION, per 100K** | **7-DAY PERSISTENCE EFFECT on SPEED**  **(# new cases per day per 100K attributed to new cases 7 days ago)** |
| --- | --- | --- | --- | --- |
| AK | 21.17 | 0.06 | -3.22 | 20.65 |
| AL | 18.15 | -1.42 | -0.82 | 23.92 |
| AR | 25.20 | 0.67 | 1.30 | 29.75 |
| AZ | 8.22 | 0.31 | -0.24 | 7.18 |
| CA | 7.63 | 0.19 | 0.34 | 9.07 |
| CO | 10.88 | 0.55 | 0.25 | 10.45 |
| CT | 8.04 | 0.77 | 1.16 | 5.54 |
| DC | 8.24 | 0.73 | 0.34 | 5.55 |
| DE | 13.13 | -0.62 | -0.65 | 13.42 |
| FL | 11.01 | 0.46 | 0.02 | 11.41 |
| GA | 10.86 | -0.06 | 0.25 | 12.14 |
| HI | 6.49 | -0.12 | -0.08 | 8.14 |
| IA | 24.95 | 1.01 | 1.78 | 27.43 |
| ID | 29.62 | 0.46 | -0.74 | 28.77 |
| IL | 17.49 | 1.01 | 0.60 | 17.63 |
| IN | 18.04 | 0.62 | -0.07 | 16.04 |
| KS | 20.61 | 0.00 | -0.61 | 24.15 |
| KY | 24.71 | -0.06 | -4.57 | 19.39 |
| LA | 15.80 | -0.08 | -4.83 | 11.92 |
| MA | 8.43 | -0.64 | -0.65 | 8.75 |
| MD | 9.25 | -0.06 | -0.17 | 9.55 |
| ME | 2.21 | -0.05 | 0.11 | 2.50 |
| MI | 10.70 | 0.58 | 0.55 | 10.40 |
| MN | 19.56 | 0.52 | -0.05 | 19.83 |
| MO | 21.52 | -0.68 | -0.03 | 24.34 |
| MS | 18.88 | -0.57 | -5.30 | 18.69 |
| MT | 42.47 | 2.47 | -2.46 | 32.87 |
| NC | 17.01 | 0.21 | -0.09 | 21.83 |
| ND | 59.35 | 2.91 | 2.01 | 56.48 |
| NE | 28.30 | 0.88 | 0.64 | 26.43 |
| NH | 5.89 | 0.28 | -0.09 | 3.12 |
| NJ | 8.46 | 1.10 | 1.43 | 7.60 |
| NM | 13.20 | 1.00 | 0.03 | 10.74 |
| NV | 15.85 | 0.23 | 0.02 | 16.23 |
| NY | 7.40 | 0.33 | 0.07 | 5.74 |
| OH | 10.94 | 0.26 | -0.16 | 10.07 |
| OK | 25.98 | 0.15 | 0.06 | 28.01 |
| OR | 7.63 | 0.44 | 0.18 | 7.37 |
| PA | 8.71 | 0.25 | 0.07 | 8.17 |
| RI | 15.25 | 1.39 | 1.55 | 8.85 |
| SC | 17.85 | 1.86 | 0.67 | 16.97 |
| SD | 52.89 | -3.91 | -14.21 | 53.44 |
| TN | 25.13 | 1.46 | 0.31 | 22.52 |
| TX | 14.43 | 0.35 | 1.12 | 15.33 |
| UT | 35.19 | 2.20 | 1.75 | 33.63 |
| VA | 11.40 | 2.33 | 2.74 | 9.53 |
| VT | 1.90 | 0.18 | 0.11 | 0.77 |
| WA | 6.92 | 0.12 | 0.15 | 7.80 |
| WI | 42.55 | 0.67 | 0.66 | 46.55 |
| WV | 10.37 | 0.08 | -0.29 | 11.44 |
| WY | 24.91 | 1.43 | 1.38 | 20.91 |
| Region | 13.85 | 0.41 | 0.13 | 14.05 |

**Table S14. Novel Surveillance Metrics for the Week of 10/12-10/18**

| **State** | **SPEED: Daily positives per 100K (weekly average of new daily cases per 100K)** | **ACCELERATION: day-to-day change in the number of positives per day, weekly average, per 100K** | **JERK: week over week change in ACCELERATION, per 100K** | **7-DAY PERSISTENCE EFFECT on SPEED**  **(# new cases per day per 100K attributed to new cases 7 days ago)** |
| --- | --- | --- | --- | --- |
| AK | 26.40 | 0.59 | 3.12 | 23.05 |
| AL | 20.94 | 1.83 | 2.29 | 19.77 |
| AR | 30.20 | 0.06 | -1.22 | 27.44 |
| AZ | 10.49 | 0.49 | -0.09 | 8.95 |
| CA | 8.53 | -0.09 | -0.05 | 8.31 |
| CO | 14.52 | -0.10 | -1.07 | 11.85 |
| CT | 9.14 | -0.87 | -1.03 | 8.75 |
| DC | 8.12 | -0.69 | -1.07 | 8.97 |
| DE | 12.88 | -0.54 | -0.35 | 14.30 |
| FL | 12.33 | 0.03 | -0.15 | 11.98 |
| GA | 11.89 | 0.50 | 0.77 | 11.83 |
| HI | 6.46 | -0.09 | 0.13 | 7.06 |
| IA | 29.76 | 0.33 | -0.11 | 27.17 |
| ID | 33.09 | -0.21 | 0.40 | 32.25 |
| IL | 23.92 | 1.08 | 0.82 | 19.04 |
| IN | 24.48 | 1.05 | 1.29 | 19.64 |
| KS | 25.51 | 0.00 | -0.24 | 22.44 |
| KY | 21.55 | 1.00 | 4.43 | 26.91 |
| LA | 11.69 | 0.67 | 4.59 | 17.20 |
| MA | 9.62 | 0.30 | 0.20 | 9.18 |
| MD | 9.74 | -0.31 | -0.58 | 10.07 |
| ME | 2.09 | -0.16 | -0.13 | 2.41 |
| MI | 14.91 | 1.51 | 0.97 | 11.65 |
| MN | 23.26 | -0.27 | -1.43 | 21.30 |
| MO | 31.19 | 0.86 | -7.01 | 23.43 |
| MS | 25.54 | 3.57 | 6.75 | 20.56 |
| MT | 56.89 | 1.46 | 3.02 | 46.24 |
| NC | 18.45 | 0.14 | -0.15 | 18.52 |
| ND | 80.03 | 3.36 | -0.56 | 64.63 |
| NE | 37.45 | 2.10 | 0.58 | 30.82 |
| NH | 5.76 | -0.01 | -0.02 | 6.42 |
| NJ | 9.40 | -0.53 | -1.22 | 9.21 |
| NM | 19.88 | 1.04 | 0.79 | 14.37 |
| NV | 19.21 | 0.81 | 0.59 | 17.26 |
| NY | 6.83 | -0.28 | -0.18 | 8.05 |
| OH | 14.15 | 0.78 | 0.03 | 11.91 |
| OK | 29.86 | 0.03 | -0.39 | 28.29 |
| OR | 8.16 | -0.40 | -0.69 | 8.31 |
| PA | 10.70 | 0.25 | 0.28 | 9.48 |
| RI | 18.78 | 0.07 | -0.26 | 16.61 |
| SC | 17.62 | 0.69 | 0.16 | 19.43 |
| SD | 74.30 | 4.72 | 7.20 | 57.59 |
| TN | 27.99 | 0.62 | 1.40 | 27.37 |
| TX | 13.80 | 0.24 | -0.03 | 15.72 |
| UT | 37.94 | -0.01 | -0.62 | 38.32 |
| VA | 12.40 | -0.86 | -1.35 | 12.42 |
| VT | 1.49 | 0.07 | 0.14 | 2.07 |
| WA | 8.07 | 0.35 | 1.08 | 7.53 |
| WI | 53.13 | 1.88 | -0.23 | 46.34 |
| WV | 14.01 | 0.62 | 0.14 | 11.29 |
| WY | 31.67 | 0.12 | -1.95 | 27.12 |
| Region | 16.03 | 0.34 | 0.11 | 15.08 |

**Table S15. Novel Surveillance Metrics for the Week of 10/19-10/25**

| **State** | **SPEED: Daily positives per 100K (weekly average of new daily cases per 100K)** | **ACCELERATION: day-to-day change in the number of positives per day, weekly average, per 100K** | **JERK: week over week change in ACCELERATION, per 100K** | **7-DAY PERSISTENCE EFFECT on SPEED**  **(# new cases per day per 100K attributed to new cases 7 days ago)** |
| --- | --- | --- | --- | --- |
| AK | 29.86 | 1.33 | 0.00 | 28.75 |
| AL | 23.02 | 3.94 | 6.22 | 22.80 |
| AR | 29.70 | -0.36 | -0.72 | 32.88 |
| AZ | 12.09 | -0.23 | -0.38 | 11.43 |
| CA | 8.07 | -0.14 | -0.52 | 9.29 |
| CO | 20.02 | 1.43 | 1.03 | 15.82 |
| CT | 13.40 | 1.34 | 0.33 | 9.95 |
| DC | 7.51 | 0.10 | 0.32 | 8.84 |
| DE | 14.20 | 0.85 | 1.16 | 14.03 |
| FL | 15.05 | 1.45 | 1.91 | 13.43 |
| GA | 12.51 | 0.20 | 0.17 | 12.94 |
| HI | 5.60 | -0.22 | -0.49 | 7.03 |
| IA | 31.28 | 0.46 | -0.14 | 32.40 |
| ID | 46.03 | 2.73 | 0.42 | 36.03 |
| IL | 32.79 | 1.05 | -0.62 | 26.05 |
| IN | 29.78 | 1.92 | 0.72 | 26.65 |
| KS | 25.99 | 0.00 | -0.96 | 27.78 |
| KY | 25.91 | 0.39 | -0.03 | 23.47 |
| LA | 15.22 | 0.09 | -1.40 | 12.73 |
| MA | 11.68 | 0.96 | 0.70 | 10.48 |
| MD | 10.47 | 0.27 | 0.46 | 10.61 |
| ME | 2.42 | 0.18 | 0.16 | 2.28 |
| MI | 18.83 | -0.36 | -0.77 | 16.23 |
| MN | 27.98 | 1.01 | 1.79 | 25.33 |
| MO | 28.33 | -0.05 | 7.41 | 33.96 |
| MS | 23.72 | -1.75 | -1.39 | 27.81 |
| MT | 62.92 | 2.74 | 2.47 | 61.95 |
| NC | 19.14 | -0.18 | -0.07 | 20.09 |
| ND | 101.21 | 3.39 | 7.20 | 87.14 |
| NE | 43.14 | -0.18 | 0.64 | 40.78 |
| NH | 5.97 | 0.00 | -0.20 | 6.27 |
| NJ | 11.89 | 0.31 | 0.16 | 10.24 |
| NM | 30.15 | 1.65 | -0.01 | 21.65 |
| NV | 22.65 | 0.62 | 0.22 | 20.92 |
| NY | 7.88 | 0.12 | -0.46 | 7.43 |
| OH | 17.83 | 0.30 | -0.10 | 15.41 |
| OK | 31.22 | 1.47 | 0.80 | 32.52 |
| OR | 7.74 | 0.01 | 0.25 | 8.89 |
| PA | 12.10 | 0.52 | 0.35 | 11.66 |
| RI | 29.07 | 2.66 | -0.98 | 20.46 |
| SC | 17.70 | -0.47 | -0.30 | 19.19 |
| SD | 80.82 | 2.84 | 7.51 | 80.91 |
| TN | 31.55 | -0.51 | -1.73 | 30.48 |
| TX | 17.39 | 0.77 | 0.48 | 15.03 |
| UT | 40.50 | 0.20 | -0.78 | 41.31 |
| VA | 11.99 | 0.00 | -0.35 | 13.50 |
| VT | 1.92 | 0.05 | -0.23 | 1.62 |
| WA | 8.19 | -0.02 | -0.94 | 8.79 |
| WI | 60.61 | -1.00 | -3.55 | 57.86 |
| WV | 15.74 | 0.47 | 0.85 | 15.25 |
| WY | 43.05 | 1.80 | -0.89 | 34.49 |
| Region | 18.39 | 0.45 | 0.24 | 17.45 |

**Table S16. Novel Surveillance Metrics for the Week of 10/26-11/1**

| **State** | **SPEED: Daily positives per 100K (weekly average of new daily cases per 100K)** | **ACCELERATION: day-to-day change in the number of positives per day, weekly average, per 100K** | **JERK: week over week change in ACCELERATION, per 100K** | **7-DAY PERSISTENCE EFFECT on SPEED**  **(# new cases per day per 100K attributed to new cases 7 days ago)** |
| --- | --- | --- | --- | --- |
| AK | 51.65 | 2.62 | -0.21 | 32.51 |
| AL | 35.21 | -3.18 | -6.88 | 25.07 |
| AR | 32.73 | -0.62 | 0.30 | 32.34 |
| AZ | 14.87 | 0.63 | 0.50 | 13.16 |
| CA | 11.63 | 0.45 | 0.16 | 8.79 |
| CO | 28.18 | 0.52 | -0.04 | 21.81 |
| CT | 20.33 | 3.27 | 2.98 | 14.60 |
| DC | 10.87 | 1.25 | 0.97 | 8.18 |
| DE | 15.04 | 0.12 | -1.25 | 15.46 |
| FL | 17.31 | -0.90 | -2.16 | 16.38 |
| GA | 15.22 | 0.05 | -0.41 | 13.62 |
| HI | 6.17 | -0.16 | 0.08 | 6.10 |
| IA | 42.37 | 2.29 | -0.44 | 34.06 |
| ID | 49.04 | -1.00 | -1.07 | 50.12 |
| IL | 41.06 | 1.60 | -0.39 | 35.71 |
| IN | 37.10 | 1.63 | -0.10 | 32.43 |
| KS | 37.21 | 0.00 | -9.22 | 28.31 |
| KY | 35.19 | 1.62 | 0.33 | 28.22 |
| LA | 13.25 | -1.16 | -0.35 | 16.57 |
| MA | 17.30 | 0.62 | -0.38 | 12.72 |
| MD | 12.78 | 0.52 | 0.06 | 11.40 |
| ME | 4.28 | 0.46 | 0.05 | 2.64 |
| MI | 28.56 | 2.73 | 0.28 | 20.50 |
| MN | 35.87 | 3.31 | 1.16 | 30.47 |
| MO | 34.84 | 2.81 | 1.25 | 30.84 |
| MS | 26.43 | 0.06 | -0.90 | 25.83 |
| MT | 69.68 | -0.55 | -0.56 | 68.51 |
| NC | 21.83 | 0.66 | 0.10 | 20.84 |
| ND | 113.94 | 6.30 | 1.09 | 110.21 |
| NE | 46.06 | 1.99 | -0.11 | 46.97 |
| NH | 8.13 | 0.53 | 0.30 | 6.50 |
| NJ | 16.34 | 0.62 | -0.40 | 12.94 |
| NM | 34.82 | -1.10 | -1.03 | 32.83 |
| NV | 26.44 | 1.33 | 1.30 | 24.67 |
| NY | 9.58 | 0.64 | 0.64 | 8.58 |
| OH | 22.62 | 1.42 | 1.13 | 19.41 |
| OK | 27.84 | -2.12 | -0.08 | 33.99 |
| OR | 10.10 | 0.67 | 0.29 | 8.43 |
| PA | 16.20 | 0.16 | -0.74 | 13.17 |
| RI | 36.65 | -1.39 | -1.73 | 31.66 |
| SC | 19.72 | -0.08 | -0.21 | 19.27 |
| SD | 112.76 | 0.44 | -10.59 | 88.01 |
| TN | 39.69 | 1.28 | 0.96 | 34.35 |
| TX | 20.55 | 0.26 | 0.08 | 18.93 |
| UT | 49.42 | 1.31 | 0.37 | 44.10 |
| VA | 13.52 | 0.16 | -0.38 | 13.05 |
| VT | 3.53 | 0.11 | 0.30 | 2.09 |
| WA | 9.13 | -0.02 | -0.09 | 8.92 |
| WI | 75.77 | 3.71 | 4.29 | 66 |
| WV | 19.20 | 0.63 | -0.51 | 17.14 |
| WY | 58.94 | 2.22 | 1.78 | 46.88 |
| Region | 22.97 | 0.63 | -0.16 | 20.03 |

**Table S17. Novel Surveillance Metrics for the Week of 11/2-11/8**

| **State** | **SPEED: Daily positives per 100K (weekly average of new daily cases per 100K)** | **ACCELERATION: day-to-day change in the number of positives per day, weekly average, per 100K** | **JERK: week over week change in ACCELERATION, per 100K** | **7-DAY PERSISTENCE EFFECT on SPEED**  **(# new cases per day per 100K attributed to new cases 7 days ago)** |
| --- | --- | --- | --- | --- |
| AK | 51.79 | -1.03 | -2.11 | 61.22 |
| AL | 29.16 | -0.18 | -1.87 | 40.93 |
| AR | 36.20 | 2.25 | 0.68 | 38.49 |
| AZ | 20.19 | 1.61 | 2.06 | 17.58 |
| CA | 11.45 | 0.14 | -0.16 | 13.69 |
| CO | 43.24 | 3.60 | 0.80 | 33.36 |
| CT | 26.50 | 1.47 | -2.63 | 24.03 |
| DC | 12.31 | -0.40 | -0.61 | 12.85 |
| DE | 17.60 | 0.85 | 1.36 | 17.75 |
| FL | 21.31 | 1.34 | 1.12 | 20.46 |
| GA | 15.50 | 0.13 | -0.16 | 17.89 |
| HI | 6.53 | 0.95 | 0.72 | 7.28 |
| IA | 71.31 | 9.29 | 7.05 | 50.10 |
| ID | 52.19 | 3.42 | 1.05 | 57.88 |
| IL | 60.40 | 4.03 | 2.42 | 48.54 |
| IN | 49.75 | 1.68 | -0.76 | 43.84 |
| KS | 49.87 | 0.00 | 1.87 | 43.60 |
| KY | 38.28 | 1.46 | 2.19 | 41.62 |
| LA | 14.22 | 0.88 | 1.13 | 15.63 |
| MA | 19.89 | 1.07 | -0.04 | 20.47 |
| MD | 15.54 | 0.56 | -0.19 | 15.11 |
| ME | 8.43 | 1.09 | 0.30 | 5.09 |
| MI | 40.37 | 2.85 | 1.70 | 33.87 |
| MN | 57.13 | 2.72 | -2.14 | 42.46 |
| MO | 43.95 | 1.15 | -0.45 | 41.25 |
| MS | 25.44 | 3.08 | 4.20 | 31.22 |
| MT | 81.74 | 1.68 | -0.67 | 82.15 |
| NC | 22.67 | -0.04 | -0.27 | 25.73 |
| ND | 163.22 | 5.87 | -0.45 | 134.57 |
| NE | 69.00 | 4.87 | 0.71 | 54.35 |
| NH | 10.93 | 1.24 | 1.19 | 9.60 |
| NJ | 20.38 | 0.66 | -0.57 | 19.38 |
| NM | 43.77 | 2.41 | -1.36 | 41.19 |
| NV | 31.57 | 0.89 | -1.41 | 31.24 |
| NY | 11.48 | 0.37 | 0.30 | 11.33 |
| OH | 32.06 | 1.68 | -0.11 | 26.74 |
| OK | 34.95 | 3.83 | 2.01 | 32.78 |
| OR | 13.70 | 0.76 | 0.19 | 11.90 |
| PA | 19.74 | 0.78 | 0.15 | 19.12 |
| RI | 46.36 | 3.51 | 2.43 | 43.10 |
| SC | 19.56 | -0.92 | -0.94 | 23.28 |
| SD | 131.62 | 5.81 | 11.19 | 132.89 |
| TN | 31.15 | -1.45 | -3.54 | 46.69 |
| TX | 23.73 | 0.87 | -1.10 | 24.26 |
| UT | 60.83 | 4.32 | 1.94 | 58.21 |
| VA | 15.09 | -0.11 | 0.21 | 15.97 |
| VT | 3.71 | 0.34 | -0.18 | 4.15 |
| WA | 12.64 | 1.41 | 1.17 | 10.80 |
| WI | 90.45 | 2.79 | -2.42 | 89.45 |
| WV | 24.56 | 1.26 | 0.97 | 22.69 |
| WY | 71.63 | 0.10 | -2.00 | 69.41 |
| Region | 28.09 | 1.24 | 0.18 | 27.11 |

**Table S18. Novel Surveillance Metrics for the Week of 11/9-11/15**

| **State** | **SPEED: Daily positives per 100K (weekly average of new daily cases per 100K)** | **ACCELERATION: day-to-day change in the number of positives per day, weekly average, per 100K** | **JERK: week over week change in ACCELERATION, per 100K** | **7-DAY PERSISTENCE EFFECT on SPEED**  **(# new cases per day per 100K attributed to new cases 7 days ago)** |
| --- | --- | --- | --- | --- |
| AK | 71.04 | 3.26 | 1.56 | 61.98 |
| AL | 33.44 | 1.80 | 1.16 | 34.90 |
| AR | 50.40 | 1.24 | -1.93 | 43.33 |
| AZ | 27.07 | -1.44 | -3.83 | 24.17 |
| CA | 17.00 | 0.85 | 0.08 | 13.70 |
| CO | 61.43 | 2.60 | -0.70 | 51.75 |
| CT | 35.42 | -2.12 | -3.08 | 31.72 |
| DC | 16.70 | 0.95 | -1.66 | 14.73 |
| DE | 26.30 | -0.22 | -2.01 | 21.07 |
| FL | 23.58 | -0.41 | -1.30 | 25.51 |
| GA | 15.91 | 0.78 | 1.08 | 18.55 |
| HI | 7.58 | -0.38 | -0.29 | 7.81 |
| IA | 112.61 | -1.55 | -10.70 | 85.35 |
| ID | 70.40 | 3.22 | 3.05 | 62.46 |
| IL | 93.34 | 3.12 | -2.65 | 72.29 |
| IN | 73.82 | 4.62 | 1.78 | 59.54 |
| KS | 83.41 | 0.00 | -13.16 | 59.68 |
| KY | 46.05 | 0.22 | -3.20 | 45.82 |
| LA | 23.62 | 9.67 | 12.80 | 17.02 |
| MA | 31.15 | 1.63 | -0.33 | 23.81 |
| MD | 23.48 | 0.66 | -1.03 | 18.60 |
| ME | 12.06 | 0.11 | 0.20 | 10.09 |
| MI | 58.53 | 1.73 | -1.45 | 48.31 |
| MN | 93.55 | 8.32 | 5.63 | 68.38 |
| MO | 67.02 | 2.44 | -0.98 | 52.60 |
| MS | 32.53 | -1.64 | -3.99 | 30.45 |
| MT | 81.04 | -0.70 | -2.34 | 97.82 |
| NC | 24.24 | 0.05 | -0.90 | 27.13 |
| ND | 175.01 | 4.95 | 6.50 | 195.35 |
| NE | 103.79 | 2.81 | -2.67 | 82.58 |
| NH | 17.46 | 0.81 | -0.39 | 13.08 |
| NJ | 31.79 | 2.46 | 1.44 | 24.39 |
| NM | 59.84 | 3.24 | 2.41 | 52.39 |
| NV | 44.15 | 0.94 | 0.76 | 37.78 |
| NY | 19.79 | 1.32 | -0.66 | 13.74 |
| OH | 48.01 | 2.62 | 0.41 | 38.37 |
| OK | 53.50 | 0.92 | -2.44 | 41.83 |
| OR | 20.46 | 1.08 | 0.16 | 16.40 |
| PA | 31.57 | 2.89 | 0.75 | 23.63 |
| RI | 67.61 | 4.85 | -0.53 | 55.49 |
| SC | 24.56 | 2.01 | 1.07 | 23.40 |
| SD | 154.46 | 10.66 | 3.79 | 157.53 |
| TN | 52.20 | 2.88 | 2.49 | 37.28 |
| TX | 28.99 | -0.07 | -0.82 | 28.41 |
| UT | 86.21 | 4.96 | 3.95 | 72.81 |
| VA | 18.12 | 0.26 | -0.47 | 18.07 |
| VT | 7.97 | 1.83 | 0.85 | 4.44 |
| WA | 16.00 | -2.76 | -4.23 | 15.13 |
| WI | 112.87 | 4.76 | 1.61 | 108.26 |
| WV | 34.68 | 1.08 | -2.83 | 29.39 |
| WY | 125.14 | 18.27 | 25.50 | 85.73 |
| Region | 39.38 | 1.45 | -0.34 | 33.62 |
