# Supplementary figures and images for: Dynamic Public Health Surveillance to Track and Mitigate the US COVID-19 Epidemic: Longitudinal Trend Analysis Study
Source: J Med Internet Res. 2020 Dec 3;22(12):e24286. doi: 10.2196/24286 (PMC7717896; doi:10.2196/24286)

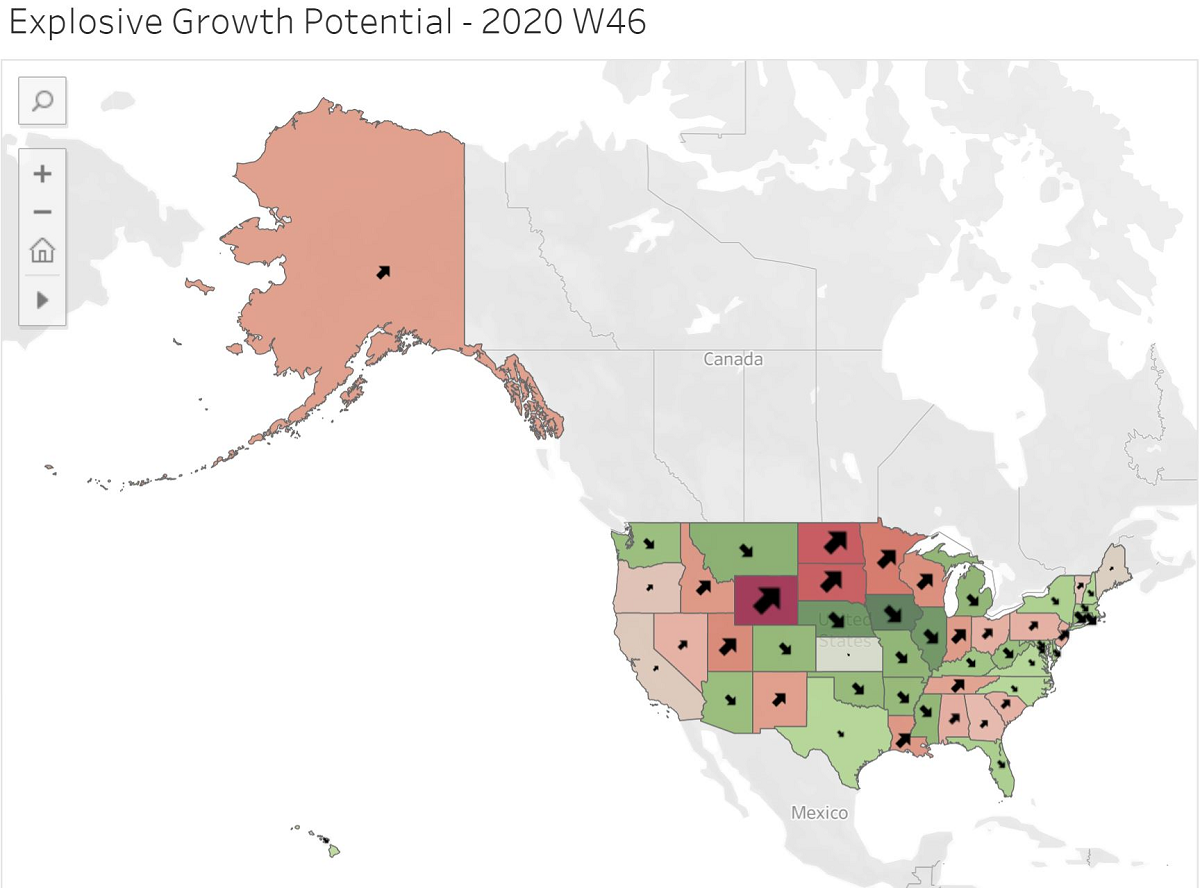

Supplement: Multimedia Appendix 2 [file jmir_v22i12e24286_app2.png]

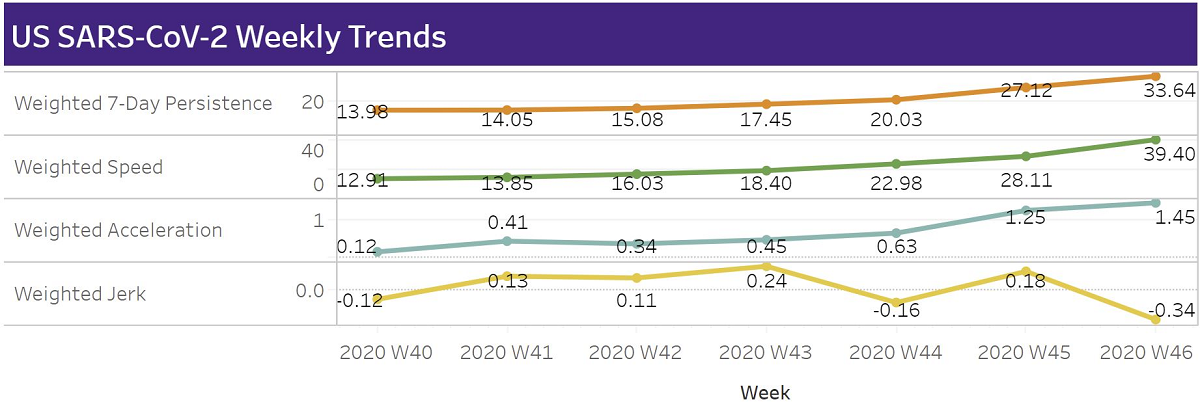

Supplement: Multimedia Appendix 3 [file jmir_v22i12e24286_app3.png]

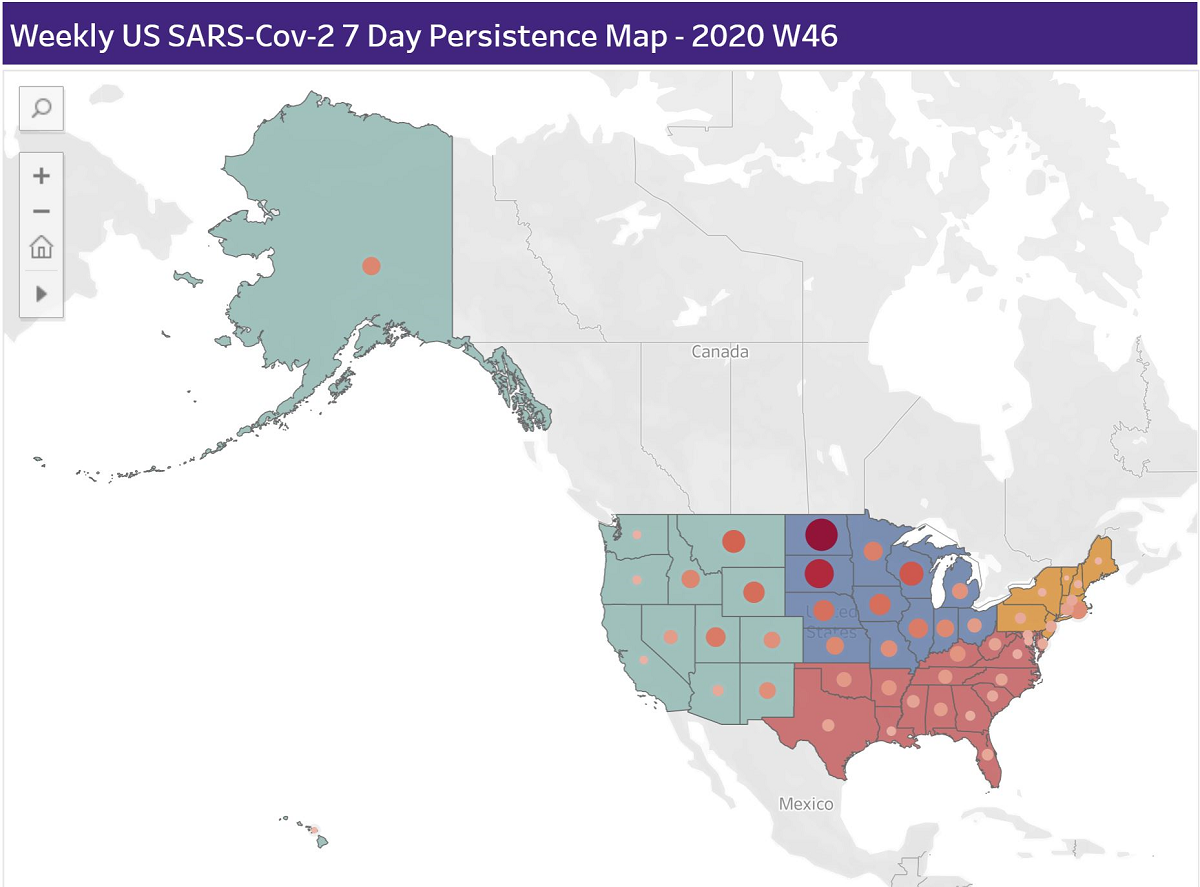

Supplement: Multimedia Appendix 4 [file jmir_v22i12e24286_app4.png]

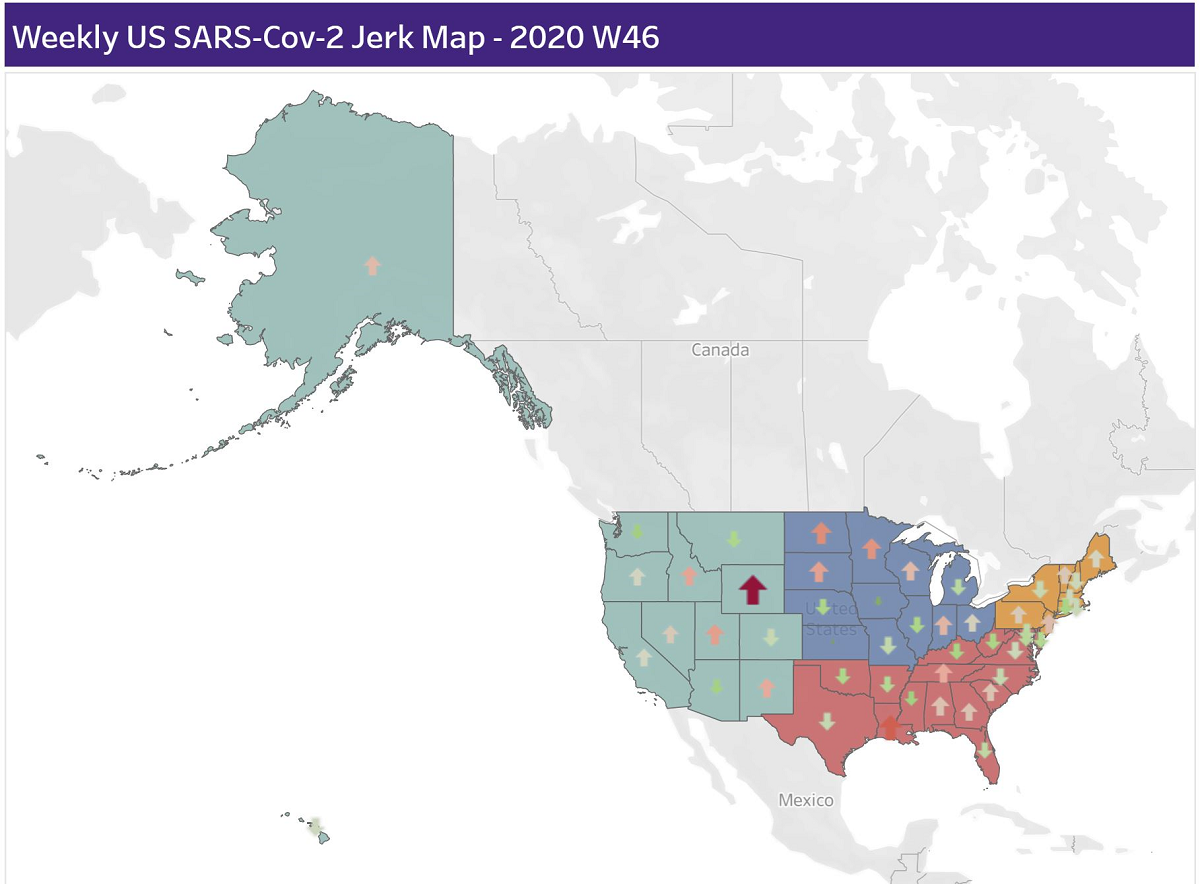

Supplement: Multimedia Appendix 5 [file jmir_v22i12e24286_app5.png]
